# Ndr kinases regulate retinal interneuron proliferation and homeostasis

**DOI:** 10.1038/s41598-018-30492-9

**Published:** 2018-08-22

**Authors:** Hélène Léger, Evelyn Santana, N. Adrian Leu, Eliot T. Smith, William A. Beltran, Gustavo D. Aguirre, Francis C. Luca

**Affiliations:** 1 Department of Biomedical Sciences, University of Pennsylvania School of Veterinary Medicine, Philadelphia, PA United States; 2 Division of Experimental Retinal Therapies, Department of Clinical Sciences and Advanced Medicine, University of Pennsylvania School of Veterinary Medicine, Philadelphia, PA United States; 3 Center for Animal Transgenesis and Germ Cell Research, University of Pennsylvania School of Veterinary Medicine, Philadelphia, PA United States

## Abstract

*Ndr2/Stk38l* encodes a protein kinase associated with the Hippo tumor suppressor pathway and is mutated in a naturally-occurring canine early retinal degeneration (erd). To elucidate the retinal functions of *Ndr2* and its paralog *Ndr1/Stk38*, we generated *Ndr1* and *Ndr2* single knockout mice. Although retinal lamination appeared normal in these mice, *Ndr* deletion caused a subset of Pax6-positive amacrine cells to proliferate in differentiated retinas, while concurrently decreasing the number of GABAergic, HuD and Pax6-positive amacrine cells. Retinal transcriptome analyses revealed that *Ndr2* deletion increased expression of neuronal stress genes and decreased expression of synaptic organization genes. Consistent with the latter, *Ndr* deletion dramatically reduced levels of Aak1, an *Ndr* substrate that regulates vesicle trafficking. Our findings indicate that Ndr kinases are important regulators of amacrine and photoreceptor cells and suggest that Ndr kinases inhibit the proliferation of a subset of terminally differentiated cells and modulate interneuron synapse function via Aak1.

## Introduction

The vertebrate retina is a complex and highly ordered neural tissue composed of strata of interconnected photoreceptors, interneurons and ganglion cells. Retinal development and maintenance require precise and coordinated regulation of gene expression, cell proliferation, cellular morphogenesis and synaptogenesis. Photoreceptors and interneurons of fully developed mammalian retinas are considered to be terminally differentiated^1–3^. The limited capacity of retinal cells to regenerate or recover in diseased or injured retinas underscores the importance of homeostatic mechanisms to maintain retinal health and function.

Defects in retinal development and maintenance cause retinal pathologies and progressive degeneration that significantly impair vision^4–6^. Recently, a naturally-occurring mutation in the *Ndr2/Stk38l* gene was shown to cause early retinal degeneration (erd) in young dogs, with disease progression accompanied by concurrent increases in photoreceptor proliferation and apoptosis, rod opsin mislocalization, progressive retinal strata disorganization and blindness^7–10^. These findings suggest that Ndr2 protein kinase is an important retinal regulator that influences the proliferative capacity of some retinal cells. Nevertheless, the precise mechanisms of Ndr2 and related kinases in retinal function remain unknown and it is unclear if mutations in *Ndr2* or *Ndr2*-related genes cause retinopathies in other species.

Nuclear Dbf2-related kinases (Ndr) belong to a subgroup of the AGC (protein kinase A, G and C) protein kinase family present in species from yeast to human, that regulate cellular proliferation and morphogenesis^11–14^. In yeasts, there are two distinct Ndr signaling pathways, the *Saccharomyces cerevisiae* Mitotic Exit Network (MEN; *Schizosaccharomyces pombe* SIN) and the *S*. *cerevisiae* Regulation of Ace2 and polarized Morphogenesis (RAM; S. pombe MOR) network)^13,15–17^. The MEN/SIN network regulates mitotic exit and cytokinesis while the RAM/ MOR network controls asymmetric gene expression, vesicle trafficking and polarized secretion^13,18–23^. In mammals, there are 4 members of the Ndr kinase subfamily, Lats1, Lats2, Ndr1 and Ndr2^17,24^. Lats1 and Lats2 are terminal kinases of the Hippo tumor suppressor pathway, which negatively regulates cell proliferation via the transcriptional regulator Yap^25,26^ and is orthologous to yeast MEN/SIN. Mammalian Ndr1 and Ndr2 kinases share ~87% amino acid sequence identity (~92% similarity) with each other and are terminal kinases in a poorly understood non-canonical Hippo pathway, referred to here as the Ndr pathway, which is orthologous to the yeast RAM/MOR signaling network^24^.

In non-retinal tissues, Ndr kinases are important regulators of cell and tissue growth^11,24^. Several studies implicate Ndr1 and Ndr2 kinases in neuronal morphogenesis, as Ndr knock down impairs neurite formation and branching while Ndr overexpression promotes neurite formation^27,28^. In brain, Ndr1/2 kinases phosphorylate the vesicle trafficking regulator Aak1 kinase and Aak1 misregulation impairs neuronal and dendritic spine morphogenesis^29^. Although the function of Aak1 in retina has not yet been ascertained, neuronal Aak1 regulates clathrin-coated vesicle (CCV) trafficking and Notch signaling^30–32^, both of which are important for retinal interneuron and Müller cell development^33,34^. Mice harboring Ndr1 or Ndr2 gene deletions are relatively healthy, but are susceptible to tumor development in skin and intestinal epithelium, consistent with a proposed role for Ndr kinases in tumor suppression^35–37^. In intestinal epithelium, Ndr1 and Ndr2 regulate epithelial cell proliferation via a Yap-dependent mechanism, suggesting that in some cells the Ndr pathway functions similarly to the canonical Hippo pathway^35^. In addition, Ndr mutations or misregulation has been linked to a variety of cancers, including breast, prostate, gastric, colorectal, lymphocytic cancers^37–39^. Ndr1/Ndr2 double KO mice are embryonic lethal at E10 and display multiple developmental phenotypes, including abnormal and displaced somites and defective cardiac development^40^. Despite these findings, the molecular functions of Ndr kinases in retinal development and homeostasis are not known.

To investigate retinal Ndr function, we developed *Ndr1* and *Ndr2* single knockout (KO) mice and analyzed structural and gene expression phenotypes of the neural retina. Here we demonstrate that deletion of either *Ndr1* or *Ndr2* causes a variety of similar phenotypes in differentiated mouse retinas, including aberrant rod opsin localization and increased cell proliferation within the inner nuclear layer (INL). Strikingly, we discovered that *Ndr1* and *Ndr2* deletion induces the proliferation of a subset of cells that express amacrine cell markers in differentiated mouse retina, while at the same time decreasing the overall number of Pax6-positive, HuD-positive and GABAergic amacrine cells. Gene enrichment analyses reveal that *Ndr2* deletion increases expression of genes associated with neuronal stress and decreases expression of genes involved in synapse maintenance/function. Consistent with these data, we demonstrate that deletion of *Ndr1* or *Ndr2* significantly decreases Aak1 protein levels in synapse-rich inner and outer plexiform layers. Taken together our data indicate that Ndr1 and Ndr2 kinases are critical regulators of retinal homeostasis and are particularly important for inhibiting amacrine cell proliferation and maintaining amacrine cell and synaptic homeostasis.

## Results

### *Ndr* KO validation

**Figure 1:**
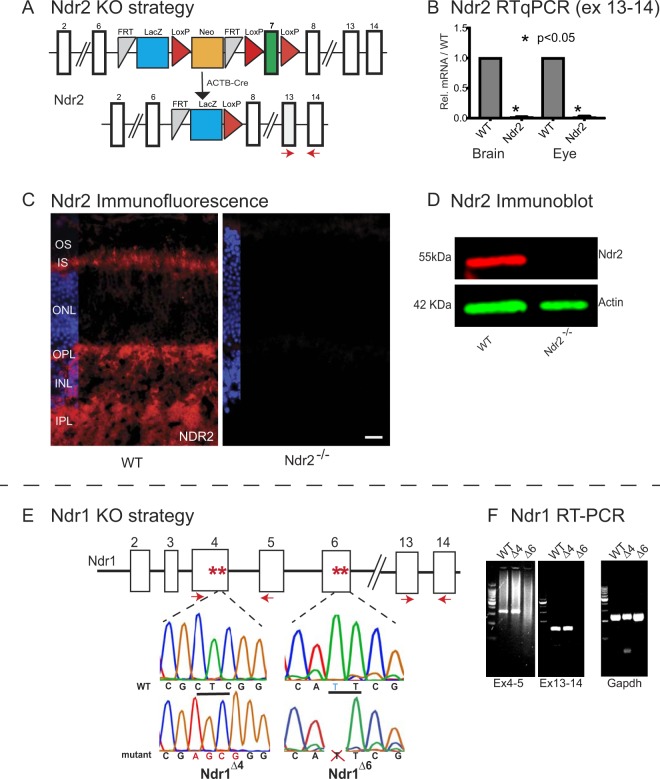
Mouse *Ndr1* and *Ndr2* knockout strategy and confirmation. (**A**) The conditional-ready *Ndr2/Stk38l* deletion allele obtained from KOMP. *Ndr2/Stk38l* exon 7 (green box) is flanked by LoxP sites (red triangles) and excised by the cre recombinase under control of the actinB promoter to produce *Ndr2* KO mice. LacZ is indicated by the blue box, Neo cassette is indicated by the orange box. RT-qPCR primers for Exons 13–14 are indicated by red arrows. (**B**) RT-qPCR data confirms *Ndr2* deletion. cDNA was isolated from brain and eye tissue from P28 wild type (WT) and *Ndr2* KO mice. Data are from 4 sets of RT-qPCRs, targeting exons 13 to 14, with each sample run in duplicate (p < 0.05, calculated by one-sample test). (**C**) Ndr2 immunofluorescence was performed on P28 WT and *Ndr2* KO retinas. Nuclei were labeled with Hoechst 33342. IPL, inner plexiform layer; INL, inner nuclear layer; OPL, outer plexiform layer; ONL, outer nuclear layer; IS, inner segments; OS, outer segments. Scale bar = 20 μm. (**D**) Immunoblot of eye protein extracts probed with anti-Ndr2 and anti-actin antibodies. Uncropped images of this immunoblot are presented in Suppl. Fig. S1. (**E**) Two independent *Ndr1/Stk38* alleles (*Ndr1*^∆4^, *Ndr1*^∆6^) were generated by distinct CRISPR-cas9 procedures and confirmed by DNA sequencing. The chromatograms of WT and *Ndr1* KO alleles are presented. The *Ndr1*^∆4^ allele contains a mutation in *Ndr1* exon 4, in which CTC (underlined) is replaced by AGCG (red) to yield a frame shift mutation. The *Ndr1*^∆6^ allele contains a single base deletion in exon 6 (blue T in WT chromatogram) to yield a frame shift mutation. Red asterisks represent stop codons introduced by indels. (**F**) RT-PCR reveal the presence of Ndr1 transcript containing exons 4–5 and exons 13–14 in retina cDNA from *Ndr1*^∆4^ and WT mice. There is no detectable Ndr1 transcript in *Ndr1*^∆6^ mice. GAPDH RT-PCR data serve as positive controls.

We generated congenic homozygous *Ndr1/Stk38* and *Ndr2/Stk38l* single KO mice to investigate the roles of Ndr kinases in retinal development and maintenance (Fig. 1, see methods). *Ndr2* was deleted in all tissues by crossing *Ndr2*/*Stk38l*^flox/flox^ mice (obtained from the Knockout Mouse Project, UC Davis), in which *Ndr2* exon 7 is flanked by loxP sites to congenic mice expressing Cre recombinase (ACTB-Cre) (Fig. 1A). The LacZ ORF within the CSD Knockout First allele is not in frame with Ndr2 exon 6, so no Ndr2-LacZ fusion protein is expected to be produced. We validated Ndr2 KO mice by PCR, DNA sequencing, immunoblot and immunohistological strategies (Figs 1B–D and S1). Although RT-PCR experiments indicated that an Ndr2 transcript containing exons 4–5 was detectable in Ndr2 KO mouse retinas, immunoblots probed with an antibody to the conserved N terminal region of Ndr1/2 revealed no evidence of truncated Ndr2 or Ndr2-LacZ fusion protein (Supp. Fig. S1C). Immunoblots probed with an Ndr2-specific antibody (generated from unique peptide sequence within the Ndr2 C-terminal region) revealed a single 55 kD immunoreactive band in wild-type (WT) mouse eye extracts that was absent from *Ndr2* KO protein extracts (Figs 1D and S1D). Likewise, comparative immunofluorescence microscopy revealed no specific Ndr2 immunoreactivity in adult *Ndr2* KO mouse retinas, whereas Ndr2 localized broadly throughout differentiated retinas of WT mice and was prominent in photoreceptor inner segments (IS), the outer plexiform layer (OPL), inner plexiform layer (IPL) and ganglion cell layer (GCL), suggesting that Ndr2 is important for the function of multiple retinal cell types (Fig. 1C).

We employed CRISPR-Cas9 methods to generate two independent *Ndr1* (Stk38) KO mouse lines (*Ndr1*^∆4^ and *Ndr1*^∆6^) that contain frame shift mutations in exons 4 and 6 (Fig. 1E). RT-PCR data indicate that Ndr1 transcripts are absent from cDNA prepared from *Ndr1*^∆6^ but not *Ndr1*^∆4^ mice (Fig. 1F). Immunoblots probed with the Ndr1/2 antibody (which recognizes both Ndr1 and Ndr2) indicated that no truncated Ndr1 protein was present in protein extracts from *Ndr1*^∆4^ (*Ndr1*−/−; Supp. Fig. S1C) or *Ndr1*^∆6^ mice (data not shown). We used both *Ndr1*^∆4^ and *Ndr1*^∆6^ KO strains throughout this study and observed no phenotypic differences between the two *Ndr1* KO lines, thereby eliminating concerns of off-target mutations or allele-specific phenotypes.

Because erd phenotypes (caused by an Ndr2 mutation) appear in young dogs after retinal neuron differentiation and lamination^7,8^, we limited our analysis of Ndr KO phenotypes to young adult mice (usually ~1 month old (P28). Ndr1 KO and Ndr2 KO mice exhibited no obvious adverse health or behavioral traits, although we did not analyze the structure and functions of non-retinal tissues. Notably, Ndr1 or Ndr2 deletion did not drastically impair vision in young mice (P28), as both Ndr1 KO and Ndr2 KO mice displayed similar ERG and visual placement responses as WT mice (Supp. Fig. S1).

### *Ndr* deletion disrupts photoreceptor homeostasis

**Figure 2:**
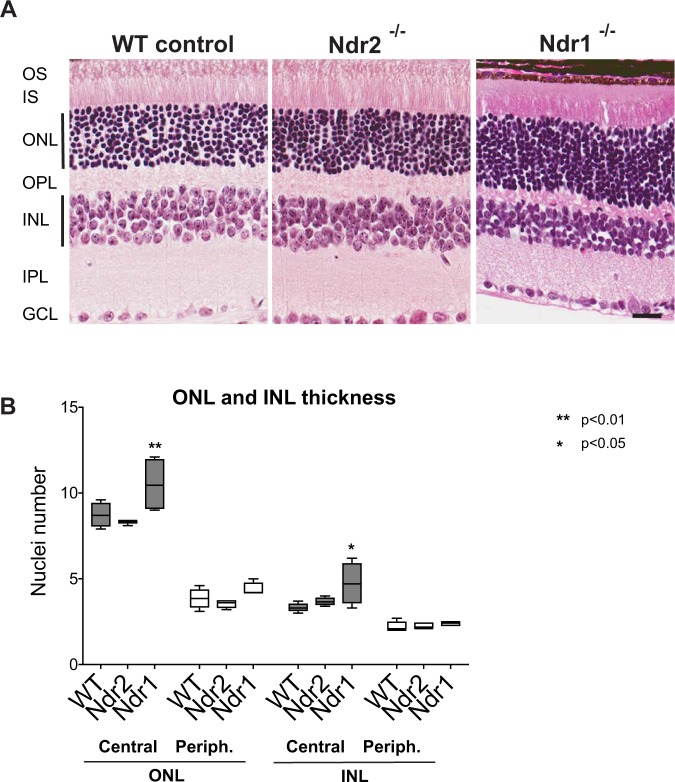
Retinal histology in adult *Ndr* KO mice. (**A**) Photomicrographs of H&E stained paraffin sections of retinas from P28 WT, *Ndr2* KO, and *Ndr1* KO mice (3 months postnatal). Scale bar, 25 μm. (**B**) Relative ONL and INL thickness of central and peripheral retina. The number of nuclei was counted manually in 10 vertical rows per section (see vertical black line), three sections per animal, n = 4 mice per genotype. *Ndr1* KO data were obtained from 2 *Ndr1*^∆4^ KO and 2 *Ndr1*^∆6^ KO mice. Means ± SD and levels of significance were calculated using an unequal variance t-test.

Since a major phenotype of canine erd retinopathy is progressive loss of retinal lamination, we analyzed retinal structure in age-matched adult *Ndr* KO and WT mice. The overall appearance and histological organization of *Ndr1* KO and *Ndr2* KO retinas were similar to that of WT retinas (Fig. 2A), observed up to 6 months of age. We compared the relative thicknesses of the outer nuclear layer (ONL) and inner nuclear layer (INL) of *Ndr2* KO and WT mice by counting the number of rows of nuclei and found no significant differences (Fig. 2B). In contrast, the ONL and INLs in the central retina of *Ndr1* KO mice were thicker than WT by ~1–3 nuclei (p < 0.05), suggesting a role for Ndr1 in photoreceptor development or homeostasis (Fig. 2B). These data indicate that *Ndr1* and *Ndr2* deletion does not significantly impair retinal strata development and organization in young adult mice. We therefore conducted immunohistochemical analyses to look for other phenotypic markers of retinal disease in *Ndr* KO mice.

**Figure 3:**
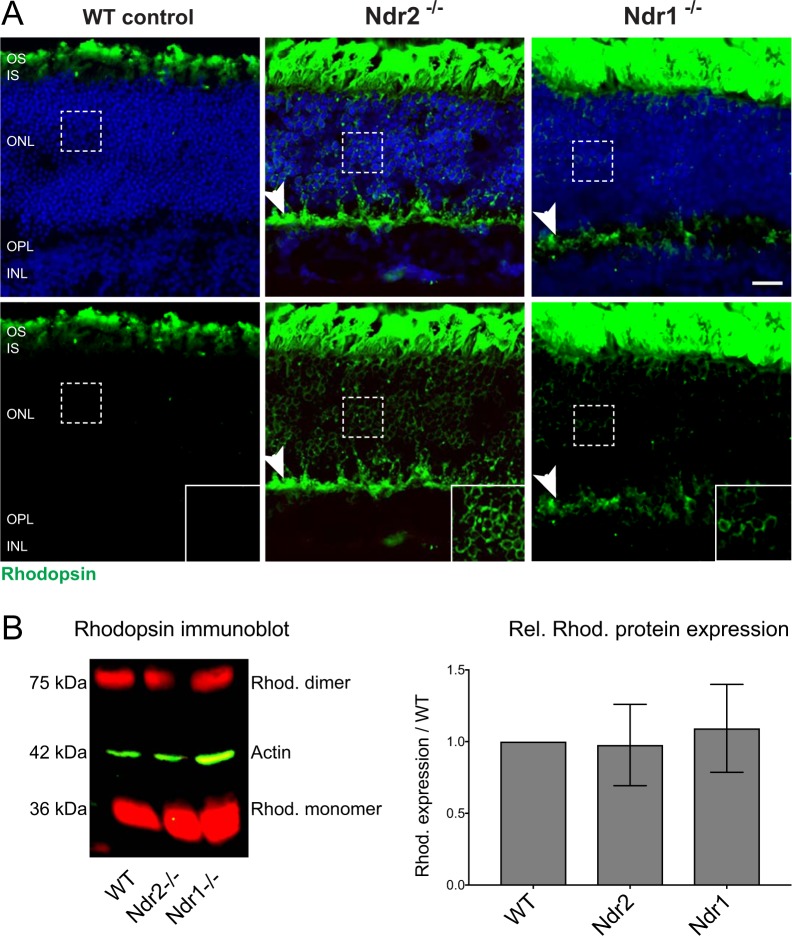
Rod opsin mislocalization in *Ndr* KO mouse retinas. (**A**) Rhodopsin immunofluorescence (green) in retinal sections from P28 WT, *Ndr2* KO and *Ndr1* KO mice. Insets show aberrant perinuclear rhodopsin localization on *Ndr* KO retinas. Arrowhead points to aberrant opsin localization in the OPL. Nuclei labeled with Hoechst 33342 (blue). Scale bar, 20 μM. (**B**) Quantitative rhodopsin immunoblot shows similar levels of rhodopsin monomer and dimers and in WT, *Ndr2* and *Ndr1* KO mice neuronal retinal extracts. Relative levels of rhodopsin monomer and dimer from 4 independent experiments were normalized to actin and plotted against WT neuronal retinal extracts. Levels of significance were calculated using a one-way ANOVA test with p < 0.05.

To determine if *Ndr1* or *Ndr2* deletion affects photoreceptors in young adult mice, we examined rod opsin localization by immunofluorescence microscopy. In fully differentiated retinas from P28 or older WT mice, rod opsin was uniformly restricted to rod outer segments (OS). In *Ndr1* and *Ndr2* KO retinas, most rod opsin localized to the rod OS, however some rod opsin conspicuously mislocalized to the inner segments (IS), the perinuclear cytoplasm in the ONL (Fig. 3A, see inset) and the rod synaptic terminals in the OPL (Fig. 3A, arrowhead).

In principle, opsin mislocalization in *Ndr* KO retinas may be caused by a variety of mechanisms, including aberrant opsin expression, post-translational processing or trafficking^41^. We conducted quantitative immunoblots on retinal protein extracts to determine if *Ndr* deletion disrupts rod opsin protein expression or electrophoretic mobility, as might be the case if Ndr influences rhodopsin glycosylation or Golgi trafficking. The relative levels and electrophoretic mobility of rhodopsin monomer and dimers were indistinguishable in *Ndr1* KO, *Ndr2* KO and WT retinal extracts (Fig. 3B), suggesting that opsin mislocalization in *Ndr* KO mice is not caused by impaired opsin expression or Golgi trafficking.

### *Ndr1* and *Ndr2* deletion promote apoptosis in the INL

**Figure 4:**
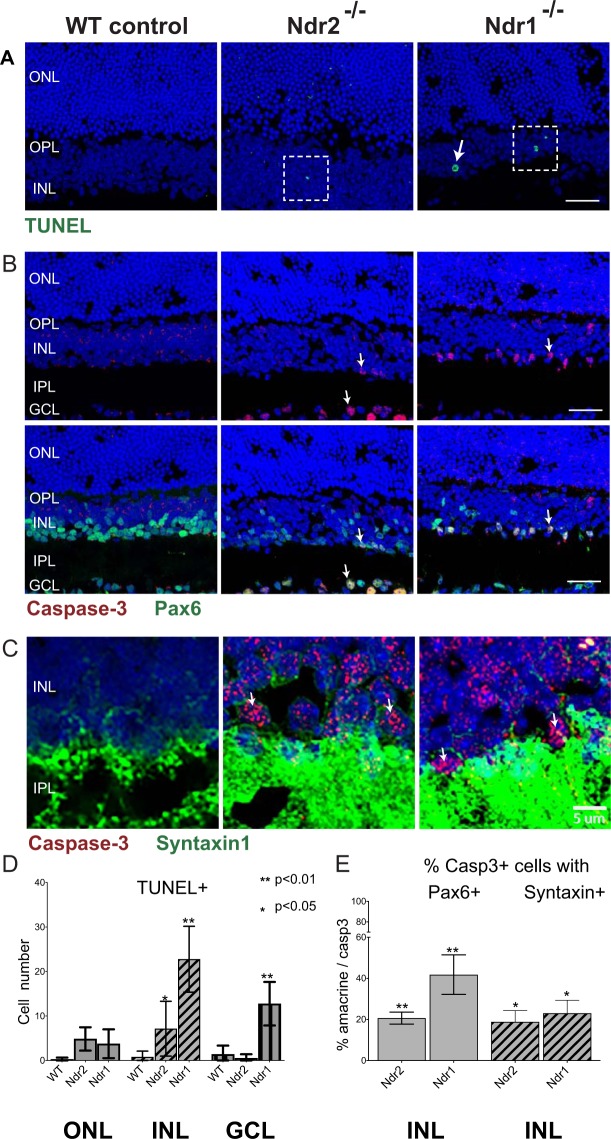
*Ndr1* and *Ndr2* deletion promote retinal cell death. (**A**) Retinas from P28 WT, *Ndr2* and *Ndr1* KO mice were labeled with TUNEL (green). Nuclei labeled with Hoechst 33342 (blue). Insets (dashed boxes) are shown in Suppl. Fig. S2A. (**B**) Active caspase-3 (red) and Pax6 (green) immunofluorescence in WT, *Ndr2* and *Ndr1* KO retinas. Arrows show representative caspase 3-positive and Pax6-positive cells. Scale bars for (**A** and **B**) 20 μm. (**C**) Active caspase-3 (red) and syntaxin (green) in WT, *Ndr2* and *Ndr1* KO retinas. Images in C were acquired by confocal microscopy and visualized as single optical sections. Scale bar, 5 μm. (**D**) The number of TUNEL-positive nuclei were counted manually in 500 μm long region of interest (ROI) and plotted; n ≥ 3 mice per genotype. (**E**) The number of active caspase-3 positive nuclei were counted manually in 500 μm ROIs and plotted; n ≥ 2 mice per genotype. Error bars represent SD and asterisks indicate levels of significance using an unequal variance t-test (p < 0.05).

To determine if *Ndr1* or *Ndr2* deletion impacts the survival of retinal cells in young adult mice, we conducted TUNEL assays to identify necrotic/apoptotic cells that contain double stranded DNA breaks (Fig. 4A,D). As expected, there were no TUNEL-positive cells in young adult (P28) WT retinas. In contrast, there were numerous TUNEL-positive cells in the INL of both *Ndr1* and *Ndr2* KO mice and in the ONL of *Ndr2* KO mice (Figs 4A,D and S2). We quantified the relative number of TUNEL-positive cells within 500 µm length of retina and found that *Ndr1* KO mice contained ~2–3 fold more TUNEL-positive cells in the INL than *Ndr2* KO mice (~6 cells/500 µm length of *Ndr2* KO INL versus ~20 cells/500 µm length *Ndr1* KO). These findings indicate that *Ndr2* deletion compromises the viability of some retinal photoreceptors (ONL) and interneurons (INL), whereas *Ndr1* deletion mainly compromises the viability of interneurons.

As a complementary method to detect retinal degeneration in *Ndr* KO mice, we analyzed the localization and expression of the apoptosis effector caspase-3 by immunofluorescence microscopy. Caspase-3 is not prominently expressed in differentiated retinas of healthy young adult mice, but is upregulated and proteolytically cleaved to an active form in apoptotic cells, in response to inherited or chemically-induced retina degenerations^42–44^. As expected, there were no active caspase 3 immunoreactive cells in mouse retinas from adult WT mice (Fig. 4B). In contrast, there were many caspase 3-positive cells in the INL and GCL of Ndr1 and Ndr2 KO retinas, indicative of apoptosis (Fig. 4B). Moreover, ~20–40% caspase 3-positive cells in the INL also express Pax6 or Syntaxin 1, suggesting that many of those cells are amacrine cells (Fig. 4B,C,E).

### *Ndr* deletion promotes cell proliferation in differentiated retinas

**Figure 5:**
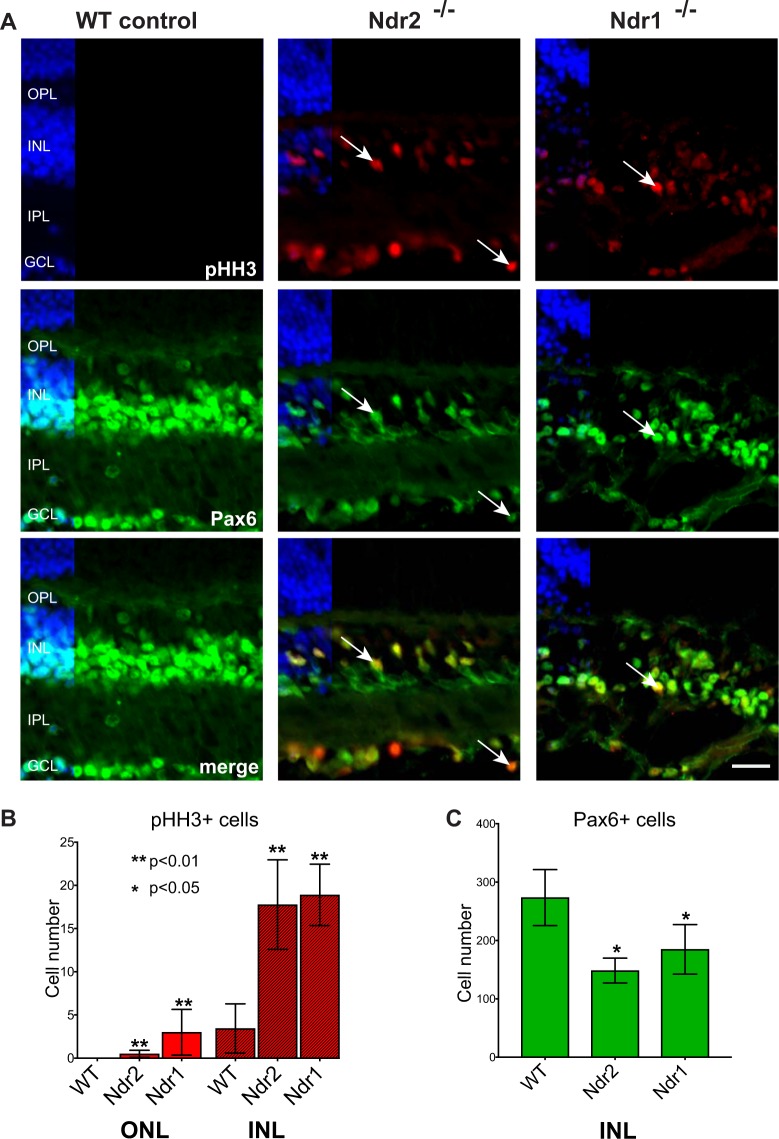
*Ndr* deletion promotes INL cell proliferation in differentiated mouse retina. (**A**) The presence of mitotic cells in P28 mouse retinas were examined in WT, *Ndr2* KO and *Ndr1* KO by phospho-histone H3 (pHH3) immunofluorescence (red). Pax6-positive cells (green) were simultaneously probed. Nuclei labeled with Hoechst 33342 (blue). Scale bar, 40 μm. (**B**) The number pHH3-positive nuclei and (**C**) Pax6-positive were quantified along 500 μm long regions of INL in ≥ 2 retinal sections per animal (n ≥ 3 animals per genotype). Error bars represent SD and asterisks indicate levels of significance using a one-way ANOVA test (p < 0.05).

The aberrant photoreceptor proliferation observed in canine erd, which is caused by an *Ndr2* mutation, suggests that Ndr kinases regulate photoreceptor proliferation^7–10,45^. To determine if *Ndr1* or *Ndr2* deletion promotes cell proliferation in differentiated mouse retinas, we probed P28 retina sections with an antibody to phospho-histone H3 S10 (pHH3), a specific marker for mitotic cells^46–48^. Fully developed mouse retinas (≥P14) do not normally contain proliferating cells, as retinal neurons and photoreceptors are terminally differentiated^8,49^. In support, there were no pHH3-positive cells in P28 WT retinas (Fig. 5A). In contrast, there were many (~17–19 cells/ 500 um length) pHH3-positive cells in the INL and GCL of *Ndr1* KO and Ndr2 KO retinas (Fig. 5B). We also detected a few pHH3-positive cells in the ONL of *Ndr1* KO mice (Fig. 5B). Parallel immunofluorescence experiments with antibodies to other cell proliferation markers, PCNA (S phase), Cyclin A (S, G2, M) and Ki67 (G1, S, G2, M phase)^50–52^ also revealed multiple proliferating cells in the INL of *Ndr1* KO and *Ndr2* KO retinas, and none in WT retinas (Supp. Fig. S3, arrows). Since most ONL nuclei belong to photoreceptors, it is likely that the mitotic (pHH3-positive) cells in the ONL of *Ndr1* KO mice are aberrantly proliferating photoreceptors, whereas the proliferating INL cells could be derived from one or more interneuron types, Müller glial cells, or retinal progenitor cells.

### *Ndr* deletion induces a subset of amacrine cells to proliferate

**Figure 6:**
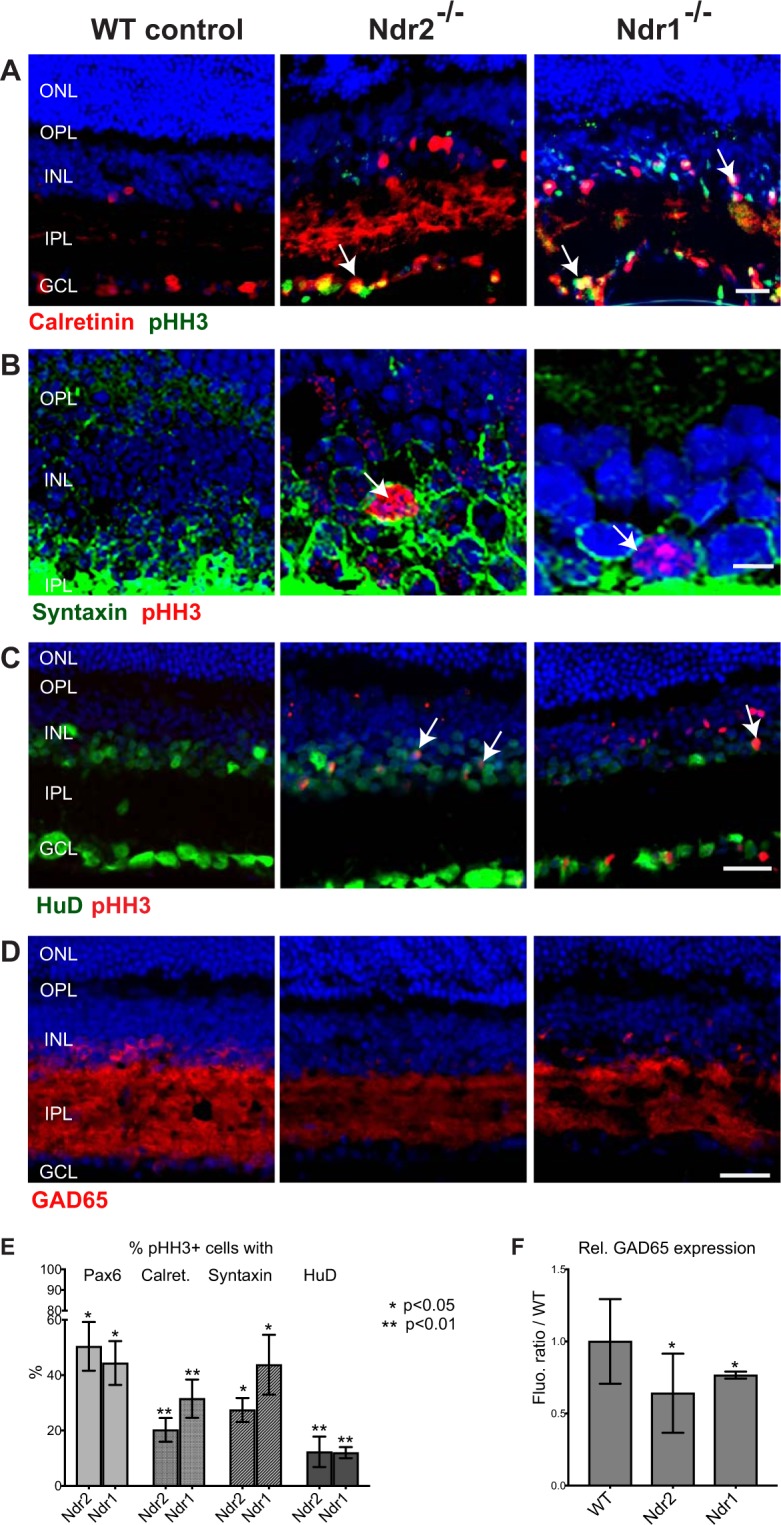
*Ndr* deletion disrupts amacrine cell homeostasis in adult mouse retina. (**A**) Immunofluorescence microscopy of the mitotic marker pHH3 (green) and calretinin (red) in P28 WT and *Ndr* KO mouse retinas. Arrows point to representative pHH3 and calretinin-positive nuclei. Scale bar, 20 μm. (**B**) Confocal immunofluorescence microscopy of pHH3 (red) and the pan-amacrine protein syntaxin 1 (green) in the basal INL of P28 WT and *Ndr* KO mice. Arrows point to pHH3-positive cell with perinuclear syntaxin 1. Images were acquired by confocal microscopy and visualized as merged z-sections. Nuclei labeled with Hoechst 33342 (blue). Scale bar, 5 μM. (**C**) pHH3 (red) and HuD (green) immunofluorescence. (**D**) GAD65 (red) immunofluorescence. Scale bar, 40 μm. (**E**) The percentage of Pax6-positive, calretinin-positive syntaxin positive and HuD-positive mitotic (pHH3) cells from 500 μm long ROIs were quantified and plotted. (**F**) Relative GAD65 immunofluorescence within the INL of *Ndr* KO retinas was quantified and plotted as ratios to WT by counting the number of pixels within five 100 μm long ROIs per retinal section (n = 3 mice per genotype). SD and statistical significance were determined by unequal variance t-test (p < 0.05).

To determine if the mitotic INL cells in *Ndr* KO retinas are amacrine, horizontal or bipolar cell interneurons, we probed retinas with antibodies to pHH3 and various cell type markers^53–55^. We observed that ~50% of the pHH3-positive cells in the INLs of *Ndr1* and *Ndr2* KO retinas expressed the pan-amacrine marker Pax6 (Figs 5A and 6E). Likewise, ~20–50% pHH3-positive cells in *Ndr1* KO and *Ndr2* KO retinas expressed three other amacrine cell markers, syntaxin 1, HuD and calretinin (Fig. 6). Many mitotic cells in the GCL of *Ndr* KO retinas also co-expressed amacrine cell markers, and these were likely normal displaced amacrine cells. In contrast, none of the pHH3-positive cells expressed the horizontal cell marker calbindin or the rod bipolar cell marker PKCα (Supp. Figs S4 and S5). These data suggest that *Ndr* deletion stimulates the proliferation of a subset of amacrine cells or cells expressing amacrine markers in differentiated retinas.

### *Ndr* deletion decreases the expression of some amacrine cell markers

Despite the abundance of proliferating Pax6-positive cells in *Ndr* KO retinas (Figs 5A,B and 6E), the total number of Pax6-positive cells is significantly diminished in *Ndr* KO retinas in comparison to WT controls (Fig. 5A,C). The relative number of Pax6+ cells in the INL is reduced by 33.7 ± 14% in *Ndr1* KO retinas and by 48.8 ± 18% in *Ndr2* KO retinas, relative to WT (p value < 0.05) (Fig. 5C). In agreement, corresponding quantitative immunoblots of normalized eye protein extracts indicate that Pax6 protein expression is diminished by ~35% (p value < 0.05) in *Ndr* KO retinas (Supp. Fig. S6).

Pax6 can be expressed in some Müller cells and retinal progenitor cells (RPC)^56,57^. Thus, we probed differentiated retinas with other amacrine markers, including HuD, Gad65 and calretinin, to determine if Ndr deletion influences amacrine cell development/maintenance. HuD is a regulator of RPC differentiation and is expressed in some mature amacrine cells^58–60^. Glutamate decarboxylase 65 (GAD65) normally localizes to neurites and nerve endings of GABAergic amacrine cells in the IPL of healthy retinas^54,55,61^ and calretinin is a calcium binding protein that is expressed in a subset of glycinergic and GABAergic amacrine cells^62^. There was a significant decrease (~60%) in HuD-positive cells in the INL of Ndr1 and Ndr2 KO retinas in comparison to WT retinas (Supp. Fig. S7). Moreover, ~10% HuD-positive cells in Ndr1 and Ndr2 KO retinas were immunoreactive to pHH3, indicating that they are proliferating (Figs 6E and S7E). GAD65 immunoreactivity decreased by ~20% in *Ndr1* KO retinas and by 40% *Ndr2* KO retinas, suggesting *Ndr* deletion reduces the abundance of GABAergic amacrine cells (Fig. 6D and F). In addition, there was a modest decrease in the number of calretinin-positive cells in the INL of *Ndr2* but not *Ndr1* KO retinas (Supp. Fig. S7). These data suggest that *Ndr* deletion reduces the abundance of mature amacrine cells, while simultaneously promoting the proliferation of some Pax6 and HuD-positive INL cells. Alternatively, *Ndr* deletion might reduce the expression of some amacrine cell markers (such as Pax6, GAD65, HuD, calretinin) without significantly diminishing amacrine cell number. Intriguingly, we also observed a modest (5–10%) increase in calbindin-positive horizontal cells in *Ndr* KO retinas, suggesting that Ndr also influences horizontal cell development (Supp. Fig. S4).

Since Pax6 and HuD are expressed in some RPCs^56–60^, it is possible that some of the proliferating Pax6 and HuD-positive cells in P28 Ndr KO retinas (Fig. 6) are progenitor cells. To test this possibility, we probed retinas for the presence of Nestin, an intermediate filament protein that is expressed prominently in RPCs and is normally excluded from mature amacrine cells. We observed a few Nestin-positive cells in P28 Ndr1 and Ndr2 KO retinas, but none in corresponding WT retinas (Supp. Fig. S8A). Notably, in Ndr KO retinas, Nestin appeared to localize to elongated cytoplasmic processes near the plexiform layers, as opposed to the perinuclear localization typical for Nestin in RPCs or developing retinal neuroblasts Supp. Fig. S8A. Since Nestin can be expressed in mature Müller glia in response to neuronal stress^63^, the aberrant Nestin expression in Ndr KO retinas may reflect neuronal stress rather than the presence of progenitor cells. Moreover, because there are far fewer Nestin-positive cells than proliferating cells in P28 Ndr KO retinas, it seems unlikely that most of the proliferating cells are progenitor cells.

### *Ndr* deletion disrupts amacrine cell interneuron organization

*Ndr* deletion appears to disrupt the cellular organization of a subset of INL interneurons. Specifically, in WT mouse retinas, most Pax6-positive amacrine cells localize prominently to the lower half of the INL with a few scattered throughout the GCL (Fig. 5), as described^64–67^. In contrast, in *Ndr* KO retinas, Pax6-positive cells in the INL was not limited to the lower half of the INL and appeared to localize throughout the INL. Moreover, in *Ndr2* KO retinas, the mitotic calretinin-positive amacrine cells localized predominantly to the GCL, whereas they localized to both the INL and GCL in *Ndr1* KO retinas (Fig. 6C), perhaps suggesting that Ndr2 influences a more limited subset of amacrine cell subtypes than Ndr1. We observed no obvious defect in horizontal or bipolar cell distribution in *Ndr* KO retinas (Supp. Figs S4 and S5).

### *Ndr* deletion does not disrupt Müller cell distribution

**Figure 7:**
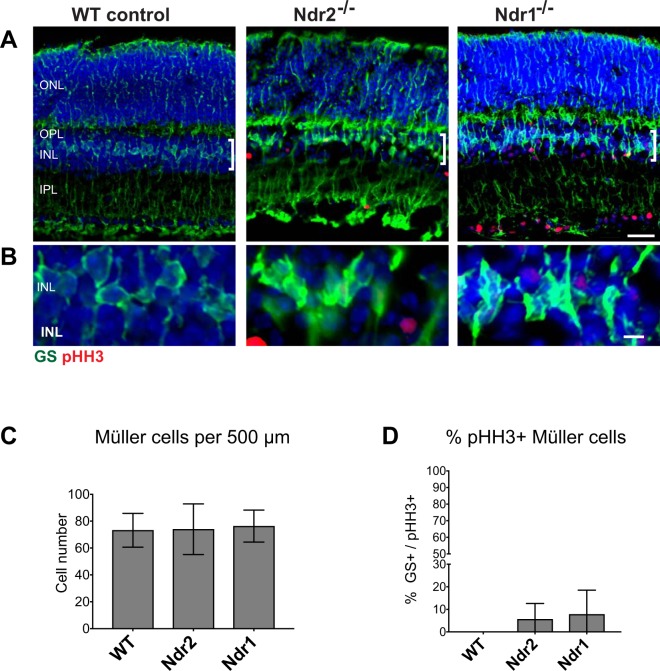
*Ndr* deletion does not significantly affect Müller cell distribution or proliferation. (**A**) Retinal sections from P28 WT, *Ndr2* and *Ndr1* KO were probed with antibodies to pHH3 (red) and the Müller cell marker glutamine synthetase (GS) (green). Scale bar, 20 μm. Brackets denote locations of Müller cell bodies (**B**). Representative images of GS-positive Müller cell bodies. (**C**) GS-positive cell bodies were quantified and plotted. (**D**) The percentage of GS-positive mitotic (pHH3-positive) cells from 500 μm long ROIs were plotted. Data were quantified from 500 μm long INL regions in ≥2 retinal sections per animal (n ≥ 3 animals per genotype). SD and levels of significance were determined using one-way ANOVA test (p < 0.05).

To determine if *Ndr1* or *Ndr2* deletion influences Müller glia proliferation or homeostasis, we simultaneously probed mouse retina sections with antibodies to pHH3 and the Müller cell marker glutamine synthetase (GS). Only a few (<8%) GS-positive cells within the INL of *Ndr1* and *Ndr* KO retinas co-expressed pHH3, suggesting that only a small percentage of Müller cells undergo proliferation in *Ndr* KO retinas (Fig. 7A,B,D). Moreover, the relative number and appearance of GS-positive Müller cells in *Ndr* KO retinas were similar to that of WT (Fig. 7C). Thus, the limited occurrence of Müller cell proliferation associated with *Ndr* deletion does not significantly alter Müller cell number or morphology in adult mice.

### *Ndr2* deletion alters neural retinal gene expression

**Figure 8:**
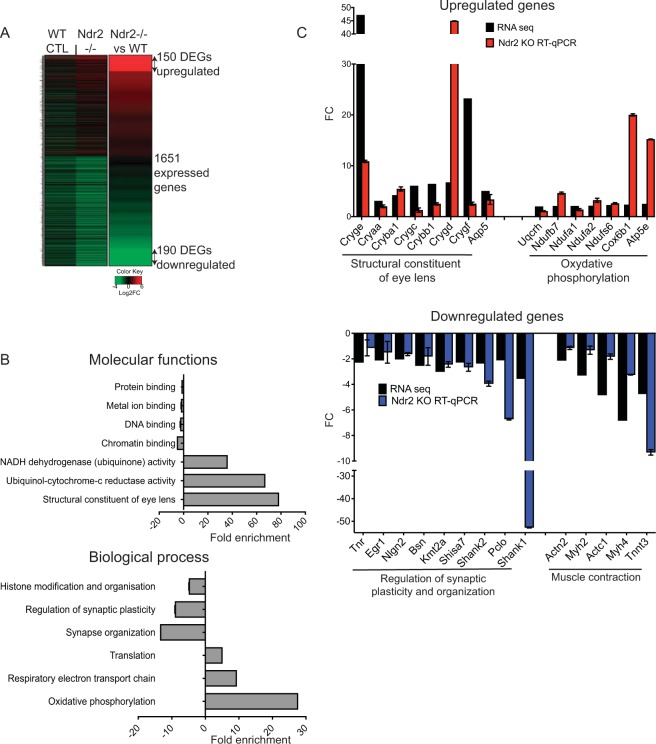
Differential gene expression in *Ndr2* KO mouse retinas identified by RNA sequencing. (**A**) Heat map showing log2 values of mRNA expression in WT and *Ndr2* KO retinas by RNA sequencing. 150 upregulated (top) and 190 downregulated (down) genes were identified in *Ndr2* KO retinas after setting a threshold to log2 ≥1. (**B**) Gene ontology enrichment for up and down regulated genes in *Ndr2* KO retina is shown for molecular function and biological process gene ontologies (p < 0.05, (FC ≥ I2I). Levels of significance were determined using a Fisher test with Bonferroni correction. (**C**) RNA seq data validation by RT-qPCR. Expression of select upregulated and down-regulated genes from the comparative RNA-seq experiments was measured by real-time quantitative PCR relative to GAPDH in mouse retina. Histograms represent the log2 fold expression calculated as 2−ΔΔCt between *Ndr2* KO and WT (log2FC ≥ I1I). Means ± SD were determined from a minimum of 2 sets of RT-qPCR experiments with each sample run in duplicate. P values were calculated by one-sample t test (p < 0.05).

To determine how *Ndr* deletion influences gene expression in fully developed retinas, we screened for genes potentially regulated by Ndr2 using RNA-seq transcriptomic profiling. We set a data threshold of ≥2 fold (log2FC ≥I1I) and identified 340 differentially expressed genes (DEGs) composed of 190 up-regulated and 150 down-regulated genes when comparing *Ndr2* retinas to that of age-matched WT retinas (Fig. 8A and Supp. Table S4). We carried out enrichment analyses on these DEGs using Database for Annotation, Visualization and Integrated Discovery (DAVID) and Gene Ontology Consortium. Functional groups (gene ontologies) for up-regulated genes included “structural constituents of eye lens”, “ubiquinol-cytochrome-c reductase activity” and “NADH dehydrogenase (quinone) activity” (Fig. 8B and Supp. Table S5). The presumed functions of many of these up-regulated genes are consistent with enhanced neuronal stress. In support, 39 of the 190 up-regulated genes are associated with the biological process gene ontologies that are commonly associated with stress, such as “oxidative phosphorylation” (cytochrome c), “respiratory electron transport chain” (NAD(P)H:quinone oxidoreductase) and “translation” (ribosomal proteins) (Fig. 8B and Supp. Table S5A,B)^68–71^. Moreover, we identified 13 crystallin genes in the top 30 up-regulated genes in *Ndr* KO neural retinas (Supp. Table S6). Crystallin proteins are known to have chaperonin and anti-apoptotic functions in stressed neuronal cells^72–74^. Among the 150 down-regulated DEGs in *Ndr* KO retinas, 18 are involved in “regulation of synaptic plasticity” and “synapse organization”, 31 are involved in “nervous system development” and 15 are involved in “histone modification and organization” (Fig. 8B and Supp. Table S5). In addition, some of the most down-regulated DEGs in *Ndr* KO retinas belong to the “muscle contraction” gene ontology (Supp. Table S5). The misregulated genes, which include actin and myosin subunits, are associated with the actin cytoskeleton and are expressed in many cell types. Cytoskeletal gene misregulation may also reflect cellular stress^75–78^.

To validate the RNA-seq findings, we conducted RT-qPCR with independently prepared mouse retinal cDNA from *Ndr2* KO and WT mice and assessed the expression of selected genes involved in the gene ontologies identified above (Fig. 8C). We also randomly selected 4 of the top differentially expressed genes (Perp, S100a6, Ckm, Atp2a1) for RT-qPCR (Table S6). RT-qPCR analysis confirmed that several genes belonging to the “structural constituent of eye lens” and the “oxidative phosphorylation” gene ontologies were up-regulated by ≥2 fold (log2FC ≥I1I) in *Ndr2* KO mouse retinas compared to WT retinas (Fig. 8C). Likewise, RT-qPCR experiments confirmed that expression of several genes from the “muscle contraction” (actin cytoskeleton) and “regulation of synaptic plasticity and organization” gene ontologies are decreased by ≥2 fold in *Ndr* KO retinas compared to WT retinas (Fig. 8C).

To determine whether Ndr1 regulates the same genes identified by the *Ndr2* KO RNA-seq screen, we conducted RT-qPCR analyses of *Ndr1* KO retinas (Supp. Fig. S9). Many genes displayed similar expression patterns in *Ndr1* KO as in *Ndr2* KO retinas. However, the expression of several genes belonging to the “synaptic plasticity and organization” gene ontology group were up-regulated in *Ndr1* KO retinas but down-regulated in *Ndr2* KO retinas. Collectively, these data suggest that *Ndr* deletion directly or indirectly induces retina neuronal stress and compromises retinal synapse and actin cytoskeletal function.

### *Ndr* deletion disrupts expression of the vesicle trafficking regulator Aak1 kinase

**Figure 9:**
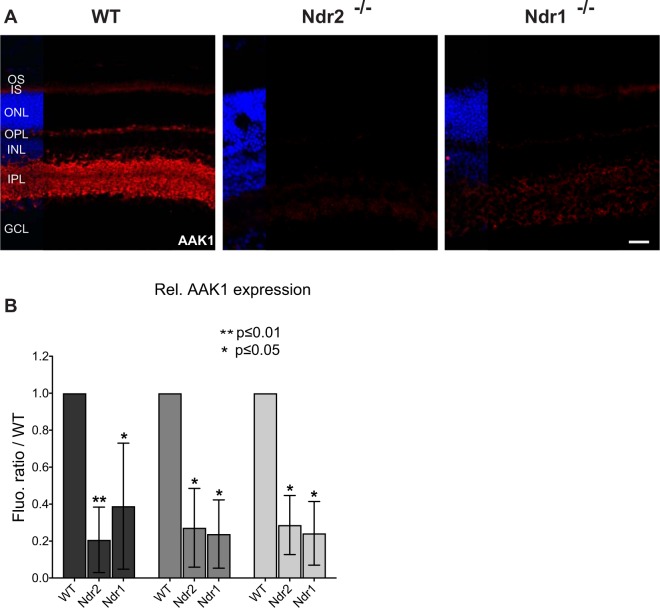
*Ndr* deletion disrupts Aak1 kinase expression. (**A**) Aak1 immunofluorescence (red) in retinal sections of P28 WT, *Ndr2* and *Ndr1* KO mice. DNA staining is shown in blue. Scale bar, 40 μm. (**B**) Relative AAK1 immunofluorescence levels were quantified by counting the number of pixels in five ROIs per retinal section (n = 3 mice per genotype). SD and levels of significance were determined by two-way ANOVA test (p < 0.01).

Previous studies indicate that Ndr kinases regulate vesicle trafficking and brain neuronal and dendritic spine morphogenesis via phosphorylation of the trafficking regulator Aak1 protein kinase^29^. We analyzed Aak1 localization in WT and *Ndr* KO mice to determine if Ndr influences retinal neuron function via Aak1. Aak1 localizes predominantly to the inner and outer plexiform layers in WT mouse retinas, consistent with a role for Aak1 in neuronal vesicle trafficking, neuronal signaling and synapse functions (Fig. 9A). In addition, Aak1 is faintly detectable in the photoreceptor inner segments (IS), suggesting a role in photoreceptor vesicle trafficking. Strikingly, there is a substantial decrease (>50%) in Aak1 immunofluorescence levels in the INL, IPL and IS of *Ndr1* and *Ndr2* KO retinas relative to WT retinas, with no concurrent increase in fluorescence in other retinal regions (Fig. 9). The same Aak1 antibody was used in control immunoblots of brain protein extracts and reveals a single immunoreactive 90 kD protein, the predicted molecular weight for Aak1. Parallel immunoblots of retinal protein extracts reveal a 50 kD immunoreactive band that decreases in abundance in Ndr KO retinas by ~30–50% relative to WT (Supp. Fig. S10). We observed the same immunolocalization and immunoblot results using two different Aak1 antibodies, thus the 50 kD immunoreactive protein likely represents a truncated form of Aak1 that may be a product of proteolytic processing or alternative slicing, as observed in other tissues^79^. RT-qPCR experiments indicate that Aak1 transcript levels are similar in Ndr KO and WT retinas (Supp. Fig. S10). Taken together with the immunofluorescence microscopy (IFM) results, these data suggest that *Ndr* deletion compromises Aak1 protein expression or stability in retinal interneurons and photoreceptors and suggest a possible mechanism for Ndr kinases in maintaining retinal interneuron, synapse and photoreceptor function.

## Discussion

### Amacrine cell regulation

*Ndr1* and *Ndr2* protein kinases can have distinct functions in some cells and can act cooperatively in others^80–83^. The similar spectrum of retinal phenotypes in young *Ndr* KO mice suggest that *Ndr1* and *Ndr2* kinases have parallel or overlapping molecular functions in the retina. Moreover, our results suggest that both *Ndr1* and *Ndr2* kinases are important for maintaining the function of multiple retinal cell types. The presence of proliferating cells in the differentiated neural retinas of adult *Ndr1* and *Ndr2* KO mice is particularly significant because most neural retinal cells are terminally differentiated^3^.

Our immunohistocytological data indicate that *Ndr1* and *Ndr2* are especially important for amacrine cell maintenance. Amacrine cells are a large class of synaptically active inhibitory interneurons that mostly reside in the INL (although some displaced amacrine cells localize to the GCL) and have dendritic arbors that project into the IPL, where they link retinal bipolar cells to retinal ganglion cells^61,84,85^. There are multiple loosely defined amacrine cell subtypes (>25, depending on reference) that are categorized by various markers, neurotransmitters and cell morphology^86^. Like other retinal interneurons, amacrine cells are considered to be terminally differentiated. Strikingly, our data suggest that *Ndr1* or *Ndr2* deletion causes a subset of amacrine cells in the INL and GCL to proliferate in differentiated retinas. At least 50% of the mitotic cells prominently express the pan-amacrine cell marker Pax6 and other amacrine cell proteins (calretinin, HuD and syntaxin 1), strongly suggesting that many of the mitotic cells are amacrine cells^53–56^ (Fig. 6). Notably, deletion of either *Ndr* gene also dramatically reduces the overall number of Pax6-positive, HuD-positive and GABAergic amacrine cells in the INL of P28 mouse retinas (Figs 5, 6 and S7). The apparent decrease in Pax6-positive, HuD-positive and GABAergic amacrine cells in *Ndr* KO mice may be a consequence of increased cell death, as suggested by elevated active caspase 3 expression, or may reflect a role for Ndr kinases in promoting cell differentiation or the expression of some amacrine cell markers (Pax6, HuD, GAD65, calretinin). Taken together, our data suggest that *Ndr1* and *Ndr2* help maintain the differentiated state of a subset of amacrine cells by preventing them from proliferating and by ensuring proper gene expression.

While our data strongly suggest that most of the mitotic cells in the INL of *Ndr* KO mice are amacrine cells, some mitotic cells do not express Pax6, suggesting that they could belong to other cell types. The Pax6-negative mitotic cells in the INL of P28 *NDR* KO mice are unlikely to be horizontal or rod bipolar cells because they fail to express markers for those cells (Supp. Figs S4 and S5)^86^. Moreover, most of the mitotic cells appear below the outer-most region of the INL, which is normally populated by horizontal and bipolar cells. A few mitotic cells express the Müller cell marker GS, suggesting they may be Müller glial cells or Müller progenitors. Intriguingly, in some non-mammalian vertebrates, such as zebrafish and *Xeonopus*, some Müller cells retain the capacity to proliferate and differentiate into other retinal cell types after retinal differentiation or in response to retinal injury^87–92^. While Müller cells are considered terminally differentiated in adult mice and other mammals^49,56,93^, recent experimental studies suggest that a small fraction of mouse Müller glial cells may reenter the cell cycle and express Pax6 in response to chemical-induced retinal injury^56,58,91,94^. Nevertheless, the Pax6-positive Müller cells from those conditions do not appear to exit S phase or label with antibodies to mitotic markers^56^. Thus, we favor the interpretation that the Pax6-positive mitotic cells in the INL of *Ndr* KO mice are amacrine cells and that most of the Pax6-negative mitotic cells are aberrant amacrine cells that fail to express Pax6. Alternatively, since Pax6 is also expressed in retinal progenitor cells (RPCs) in developing retinas, it is possible that some of the Pax6-positive mitotic cells in differentiated *Ndr* KO retinas are RPCs or amacrine cell progenitors.

The origin and fate of proliferating cells in the INL of adult Ndr KO retinas are not known. In principle, the proliferating cells may be derived from RPCs that persist and proliferate after the normal period of RPC proliferation in developing retinas. Alternatively, some developing amacrine cells in Ndr KO retinas may be cell cycle delayed or arrested in the retinal INL of young adult Ndr KO mice, thereby leading to the appearance of actively proliferating cells in differentiated retinas. Although we have not ruled out those possibilities, we do not favor either of those explanations. Notably, the proliferating cells appear to be devoid of the RPC marker Nestin and there are fewer HuD-positive cells in Ndr KO than in WT retinas, suggesting that the proliferating cells are not derived from canonical RPCs. Moreover, it seems unlikely that Ndr deletion leads to cell cycle arrest in the developing retina, as the failure to complete cell division would likely result in decreased INL thickness and induce more robust apoptosis and retinal degeneration than observed. A third possibility is that some of the proliferating cells may be derived from differentiated or nearly differentiated amacrine cells. Intriguingly, a subset of amacrine cells in differentiated mouse retinas express the stem cell marker Lgr5, a receptor for Wnt family ligands^95,96^. As Lgr5-positive amacrine cells may be capable of proliferating and differentiating into other interneuron cell types, including photoreceptors^95,96^, it is tempting to speculate that the mitotic Pax6-positive mitotic cells that we observe in the INLs of adult Nrd KO mice are derived from a similar pool of proliferation-competent amacrine cells. If so, Ndr signaling may function to help maintain the balance between amacrine cell proliferation and differentiation. Since there is no significant change in retinal thickness in young adult Ndr KO mice, our data suggest that there is a balance between INL cell proliferation and cell death in Ndr KO retinas. Indeed, misregulation of some cell cycle regulators can lead to increased apoptosis^81,97–99^. Thus, some of the proliferating INL cells in *NDR* KO retinas may ultimately become apoptotic, while others may have amacrine cell fates. Further studies using methods to disrupt Ndr function in mature retinas are needed to address the role for Ndr in maintaining retinal cell fate and differentiation.

### Role of *Ndr* in cell proliferation

Our data suggest that mouse Ndr1 and Ndr2 inhibit amacrine cell proliferation in differentiated retina, although the responsible molecular mechanisms remain unknown. Numerous studies link the Ndr subfamily of protein kinases, especially Lats1/2 kinases, to regulation of cell proliferation^83^. The role of Ndr-related Lats1/2 kinases, which are terminal kinases in the canonical Hippo tumor suppressor pathway, in regulation of cell proliferation is well established. Lats1/2 kinases inhibit proliferation by phosphorylating the transcription activator Yap^100^, thereby preventing Yap nuclear translocation and Yap-dependent expression of cell cycle and anti-apoptosis genes^83,101^. Consequently, loss-of-function alleles of Lats and other Hippo pathway components cause tissue overgrowth^83^. It is possible that Ndr1 and Ndr2, which are related to Lats1/2 kinases, negatively regulate retinal cell proliferation via similar Yap-dependent mechanisms. In support, Ndr1/Ndr2 kinases inhibit intestinal epithelial cell proliferation via a Yap phosphorylation^35^.

Yap is an important regulator of early retinal development, as it controls RPC proliferation and differentiation and influences RPE development^102,103^. Recently, Yap was shown to be expressed in a subset of retinal Müller cells in adult mouse retinas^104^. However, *Ndr1* and *Ndr2* deletion does not appear to significantly influence Müller cell proliferation or abundance in adult mice, suggesting that Ndr does not inhibit Müller cell proliferation via Yap phosphorylation. Moreover, we did not observe any significant change in Yap protein localization in the INL of differentiated retinas from P28 *Ndr* KO mice (unpublished data). These data argue against the model that Ndr kinases inhibit Müller cell and amacrine cell proliferation via Yap phosphorylation. Nevertheless, the remaining Ndr kinase in single *Ndr* KO mouse may impede detection of robust Yap-related phenotypes, thus it may be necessary to delete both *Ndr1* and *Ndr2* genes to definitively test this model. Alternatively, since Ndr1 and Ndr2 may negatively regulate amacrine cell proliferation via indirect mechanisms or via another, as yet unidentified, substrate, further analyses of putative Ndr substrates will be necessary to elucidate the precise molecular mechanisms of Ndr in modulating amacrine cell proliferation.

### *Ndr* and amacrine cell homeostasis

Despite an apparent increase in amacrine cell proliferation in differentiated retinas of *Ndr* KO mice, there were fewer Pax6-positive and HuD-positive cells and significantly less Gad65 expression in *Ndr* KO retinas than in WT retinas. Pax6 is a pan-amacrine cell marker and is essential for RPC multipotency and amacrine cell differentiation^57^ and HuD is a regulator of RPC differentiation and is expressed in some mature amacrine cells^58–60^. Thus, these data suggest that Ndr promotes amacrine cell development and/or maintenance. While a decrease in amacrine cell differentiation in *Ndr* KO retinas could potentially cause a correlative increase in development of other INL cell types, we found no evidence for significant changes in relative number or distribution of Müller, horizontal or bipolar cells (Figs 7, S4 and S5, and data not shown) in the INL of *Ndr* KO mice. Moreover, despite the decrease in Pax6, HuD and Gad65-positive cells in *Ndr2* KO retinas, the relative INL thickness (measured by nuclei) of the central retina is similar to WT controls, arguing against a precipitous loss of amacrine cells. Thus, we favor the interpretation that *Ndr* deletion does not decrease the overall number of amacrine cells, but instead alters the cellular physiology of a subset of amacrine cells, leading to decreased expression of some amacrine cell proteins, such as Pax6, HuD and Gad65. Moreover, since Gad65 is marker for GABAergic amacrine cells^105^, the reduction in Gad65 immunoreactivity in *Ndr* KO retinas suggest that Ndr1 and Ndr2 are important for maintaining the proper balance of amacrine cell subtypes within the INL, perhaps via a combination of transcriptional and posttranscriptional mechanisms.

### *Ndr* and retinal neuron stress

Our gene expression data suggest that Ndr2 deletion induces neuronal stress. In support, *Ndr2* deletion is accompanied by increased expression of genes associated with oxidative stress (such as the ROS scavenging enzyme NAD(P)H quinone oxidoreductase), mitochondrial dysfunction (cytochrome c proteins), protein misfolding (crystallins) and cytoskeleton misregulation (actin and myosin) (Supp. Table S5). Intriguingly, many of these classes of genes are affected in retina after toxic injury, such as methanol intoxication^106^, further supporting a role for Ndr2 in maintaining retinal homeostasis^107^. Oxidative and mitochondrial stress often arise from an imbalance between mechanisms that generate reactive oxygen species (ROS) and cellular detoxification mechanisms, and can lead to neuronal dysfunction and cell death^108,109^. In retina, ROS is commonly generated by light-induced signal transduction pathways, oxidization of polyunsaturated fatty acids, and RPE-mediated phagocytosis of photoreceptor outer segments^108,109^. Healthy retinal neurons maintain homeostasis under conditions of moderate OS, however an aberrant increase in ROS caused by constant exposure to light or mislocalized opsin can lead to activation of caspase-3 and apoptotic pathways causing cell death and visual impairment^110,111^.

Although the mechanism for Ndr kinases in preventing neuronal stress is not known, Ndr loss-of-function may indirectly promote neuronal stress via a variety of methods, including opsin misclocalization, impaired gene expression, neuronal dysfunction, increased apoptosis, or aberrant vesicle trafficking/synapse functions. Intriguingly, previous studies demonstrate that in presence of light, rhodopsin mislocalization to the ONL can cause oxidative stress that is not compensated by the RPE cells. Thus, it is possible that in Ndr KO mice, the mislocalized rhodopsin in the ONL and OPL might contribute to elevated ROS production and a correlative increased expression of ROS scavenging enzymes, such as NAD(P)H:quinone oxidoreductase. Alternatively, elevated neuronal stress in Ndr KO retinas may be an indirect consequence of misregulated interneuron homeostasis and increased apoptosis in the INL.

### *Ndr* and synapse regulation

Our gene expression analyses reveal that *Ndr2* deletion decreases expression of genes involved in synapse function and modulation. These data suggest that Ndr directly or indirectly regulates interneuron neurite and synapse function. In agreement, Ndr2 prominently localizes to the synapse-rich inner and outer plexiform layers. Moreover, previous studies in several diverse organisms implicate Ndr kinases in regulating neuronal morphogenesis^17,112^. Notably, mutations in *Drosophila* and *C*. *elegans* Ndr kinase homologs (Trc and Sax-1) cause defects in neuronal tiling and dendritic spine morphology and overexpression of Ndr kinases promote neurite formation and branching in cultured cells^113,114^. Yeast Ndr also regulates vesicle trafficking, polarized secretion and morphogenesis^12–14,20,24,115^, which are essential for neuronal cell development and function^116–118^.

It is likely that retinal Ndr kinases regulate interneuron function by modulating vesicle trafficking. In support, mammalian Ndr2 influences integrin trafficking and integrin-dependent neurite growth in hippocampal neurons^28^. Yeast Ndr regulates trafficking of secretory vesicles via phosphorylation of Sec2/Rabin8, a GEF for a Rab GTPase^20^. In mouse, Ndr1 and Ndr2 phosphorylate Rabin8 and Aak1 protein kinase, both of which are involved in neuronal vesicle trafficking^29,119^. Mutations that disrupt Aak1 phosphorylation cause neuronal branching and dendritic spine defects in hippocampal cell cultures, supporting a role for Ndr and Aak1 in and neuronal morphogenesis and synapse function^29^.

Aak1 kinase function is not fully understood and has not been investigated in retina. Previous studies indicate that Aak1 regulates clathrin coated vesicle trafficking during endocytosis via phosphorylation of the AP2 adapter complex and regulates trafficking of components involved in the Notch-signaling pathway, which is important for amacrine and Müller cell fate specification and retinal development^32–34,120,121^. We demonstrated that Aak1 localizes to the synapse-rich plexiform layers and is significantly diminished in *Ndr1* and *Ndr2* KO mutants. Thus, we hypothesize that retinal Ndr kinases modulate interneuron vesicle trafficking and synapse function via Aak1. Intriguingly, both Ndr1 and Ndr2 kinases are required to maintain WT levels of retinal Aak1. Thus, loss of Ndr signaling might impair retinal interneuron vesicle trafficking and neuronal signaling in retinas via diminished Aak1 levels/activity.

### *Ndr* KO mice as a model for erd

Many retinal phenotypes in *Ndr* KO mice are shared with canine erd, including rod opsin mislocalization, increased cell proliferation and apoptosis, and impaired gene expression^7,8,10^. However, the progressive loss and disorganization of ONL in erd dogs suggests that erd more severely compromises photoreceptor integrity than does mouse *Ndr1* or *Ndr2* deletion. In addition, the rod opsin mislocalization phenotype and presence of TUNEL-positive cells and mitotic cells within the ONL suggest that photoreceptors are stressed by *Ndr* deletion. *Ndr* deletion also appears to disrupt the organization of Pax6-positive amacrine cells (Fig. 5A) and increase the fragility of fixed retinas (data not shown), further implicating Ndr kinases in maintenance of retinal structure and organization.

The differences between canine erd and mouse *Ndr* KO phenotypes may be attributed to a number of factors, including species-dependent differences in retinal homeostasis or Ndr function, the presence or absence of as yet unidentified genetic modifiers, intrinsic differences between mutant *Ndr* alleles, and differences in the relative importance of each *Ndr* gene with respect to specific cell types or functions. Notably, it has not been determined if the mutant canine Ndr2 erd allele is a complete loss-of-function allele or if it encodes a catalytically inactive truncated Ndr2 protein. The canine Ndr2-erd allele contains a 4 nt deletion in intron 3 and an exonic SINE insertion, leading to the deletion of the exon 4 from the canine *STK38L* transcripts^7^. This mutation could, in principle, could result in the expression of mutant Ndr2 protein that lacks amino acids encoded by exon 4. Thus, the differences in the mouse Ndr2 KO and canine erd alleles may account for phenotypic differences. It is also possible that there are species-specific differences in Ndr function or retinal development and maintenance mechanisms that account for the apparent phenotypic differences in mice and dogs. Regardless of differences between canine erd and mouse *Ndr* KO phenotypes, our experiments reveal that Ndr1 and Ndr2 are important regulators of retinal interneurons and broaden an understanding of retinal Ndr kinases. Additional studies are needed to further elucidate the shared and cell-type specific functions of Ndr1 and Ndr2 kinases in retinal development and disease.

## Materials and Methods

### Animal care IACUC compliancy

All mice were treated in accordance with the Federal Guide for the Care and Use of Laboratory Animals and in accordance with the ARVO Statement for the Use of Animals in Ophthalmic and Vision Research; all protocols were approved by the animal care review board of the University of Pennsylvania (protocol #805078). Mice were raised in group-housing polypropylene cages at 12-h light/dark cycle with ad libitum access to food and water. For most of the experiments, 1-month animals (P28) were used unless otherwise indicated. All efforts were made to minimize the number of animals used and their suffering. An equal percentage of male and female mice were used for each of the described experiments.

### Generation of *Ndr* KO mice and validation

The *Stk38l/Ndr2* mouse strain (KOMP #71464) used for this research project was created from ES cell clone EPD0396_5_A05 generated by the Wellcome Trust Sanger Institute and introduced into mice by the KOMP Repository (www.komp.org) and the Mouse Biology Program (www.mousebiology.org) at the University of California Davis^122^. The *Stk38ltm1a*(*KOMP*)*Wtsi* conditional-ready knock-out allele contains three loxp sites flanking a neo expression cassette and *Ndr2/Stk38L* exon 7 (Fig. 1A) encoding a portion of the kinase domain. To inactivate *Ndr2* in all tissues, *Stk38L*^*flox/flox*^ mice were mated to *FVB/N-Tg*(*ACTB-cre*)*2Mrt/J* mice (Jax lab #003376), in which Cre recombinase is driven by the human beta Actin gene promoter and is expressed in all mouse tissues by the blastocyst stage of development^123^. *Ndr2* KO mice were backcrossed more than eight generations with C57BL/6J mice (Jackson Laboratory #003376). Genotypes were determined by PCR amplification of tail genomic DNA (See Supp. Table S1 for oligos). To investigate Ndr2 mRNA levels in WT and Ndr KO mice, RT-PCR or RT-qPCR was conducted to probe mouse eye and retina cDNA for transcripts containing Ndr2 exon 7, transcripts spanning exon 3–6 and transcripts spanning exon 13–14 (See Supp. Table S3 for oligos). All *Ndr2* KO mice are viable, fertile and healthy.

### Generation of Ndr1/STK38 (Ndr1) KO mice, selection and validation

Two independent *Ndr1/Stk38* KO mouse lines were generated by introduction of frame shift mutations in *Stk38* exons 4 and 6 by CRISPR/Cas9 gene-targeting methods, as described^124,125^. Single-guide RNA (sgRNA) to *Ndr1* exons 4 and 6 (Supp. Table S1) were designed using the CRISPR design tool CHOPCHOP (http://chopchop.cbu.uib.no/index.php)^126,127^. To generate templates for sgRNA transcription, gene-specific oligonucleotides containing a T7 promoter sequence, a 20 bases target-specific site and a complementary region were annealed to a constant oligonucleotide encoding the reverse-complement of the tracrRNA tail (Supp. Table S1). The ssDNA overhangs were filled in with T4 DNA polymerase (NEB #M0203S), the resulting sgRNA template were purified (DNA clean concentrator kit, Zymo # D4003) and transcribed (Ambion™ MEGAshortscript™ kit T7, #AM1354). Purified linear sgRNA and Cas9 protein (University of Utah Mutation Generation and Detection Core) were mixed in 40 µL nuclease-free microinjection buffer (5 mM Tris-HCl, pH 7.4, 0.1 mM EDTA, pH 8.0) to final concentration of 50 ng/µL sgRNA and 100 ng/µL of Cas9 and microinjected into C57BL/6 mouse zygotes (Center for Animal Transgenesis and Germ Cell Research, University of Pennsylvania).

HRMT assay and Sanger sequencing (Fig. 1E) were used to identify insertion/deletion (Indels) caused by non-homologous end joining repair at the cut site in *Ndr1* exon 4 (*Stk38*^∆4^) and exon 6 (*Stk38*^∆6^). Since mutant mice generated by zygote injection are frequently mosaic, mutant founder *Ndr1* animals were outcrossed to C57BL/6J mice to establish stable and uniform transgenic *Ndr1* KO lines. *Ndr1*^∆4^ mice were genotyped by Kompetitive Allele Specific PCR (KASP) (LGC Genomics) according to manufacturer’s protocols^128^. *Ndr1*^∆6^ were genotyped using real time PCR with specific probes designed by Transnetyx (Cordova, TN; project 1773.24; Ndr1_i3e4ie_M2). To validate Ndr1 deletion and compare relative Ndr1 transcript levels in WT and Ndr1 KO mice, RT-PCR was conducted to probe mouse retinal cDNA for transcripts that span Ndr1 exon 4–5 and exon 13–14 (see Supp. Table S3 for oligos). Both *Ndr1* KO mouse strains are viable, healthy and fertile and display no obvious gross phenotype.

### Visual function assessment

Visual placement response assays were performed on ≥3 animals per genotype (1–2 months old), as in^129^. Briefly, mice are held 25 cm above a clean surface and assayed for limb extension as animals are lowered toward the surface. Numbers were attributed for each of the following behavioral responses: 0 = no response, 1 = response upon nose contact, 2 = response upon vibrasse contact, 3 = response before vibrasse contact ≥18 mm. Normal mice reach their forelimbs toward the impending clean surface (scoring = 3), while blind animals will not (scoring 1–2). Ndr1 KO and Ndr2 KO mice scored indistinguishably from wild type mice (scoring = 3). Electroretinography (ERG) recordings were performed on ≥3 animals per genotype (1–4 months old) by the Noninvasive Assessment of Visual Function Facility (Penn Vision Research Center supported by the NIH core grant P30 EY001583), as in^130^.

### Protein extract preparation and Immunoblot analysis

Brain, eyes or retinas from ≥three animals were homogenized in cold RIPA buffer containing proteases inhibitors (Roche Complete Mini-EDTA free protease inhibitors, 100 μM leupeptin, 100 mM NaVO4, 20 mM NaF) using a BeadBug™ Microtube Homogenizer (Benchmark Scientific Model D1030E) as in^10^. 50 μg extract samples were electrophoresed on 4–12% SDS- polyacrylamide gels and transferred to nitrocellulose membranes (Li-COR #926–31090). Membranes were incubated in Odyssey® Blocking Buffer (Li-COR, #P/N 927–50000) for 1 h at room temperature, treated with primary antibody (Supp. Table S2) in Blocking Buffer +0.1% Tween 20 for 12–6 h at 4 °C and incubated for 1 h at room temperature in secondary antibody (goat anti-rabbit IRDye 800CW or goat anti-mouse IRDye680RD; LiCOR). Membranes were scanned on Odyssey Fc Dual-Mode Imaging System (Li-COR) and data quantified using Image Studio Software (Li-COR), according to manufacturer’s protocols. Actin was used as a reference protein for quantitative immunoblots. All commercially available antibodies used for immunoblots and immunofluorescence experiments are listed in Supp. Table S2. Affinity purified rabbit polyclonal anti-Ndr2 antibody was generated using an Ndr2-specific peptide antigen (QPVPNTTEPDYKSK, corresponding to amino acids 421–434) (YenZym Antibodies LLC, San Francisco, CA), as previously described^29^.

### Histological analysis and quantification

For histological analysis, right eyes from 1 month to 3 months old mice (n ≥ 4) were harvested, fixed in Excalibur alcoholic solution, a modified Davidson solution (Paula Pierce, Excalibur Pathology Inc.), paraffin embedded and sectioned (5 um). Retinal tissues were deparaffinized and counterstained with Hematoxylin and Eosin (H&E)^10^. To measure retinal thickness, H&E retinal sections were scanned and digitized using the Aperio scanscope CS-OT (Leica) and visualized using Aperio Image Scope software. Three retinal sections per mouse were used for quantitative evaluation of ONL and INL thickness, measured as the number of rows of nuclei at specific locations (central = optic nerve head (ONH) (±100 µm) and peripheral = ONH ±2000 µm (±100 µm)). The number of nuclei were counted and averaged, as in^131^.

### Immunohistochemistry and analysis

For immunohistochemistry, left eyes (n ≥ 3 per assay) were fixed in 4% paraformaldehyde in PBS for 15 minutes on ice, incubated overnight in PBS containing 15% and 30% sucrose and embedded in optimal cutting temperature (OCT) compound. 10 μm frozen sections were air-dried at RT and immunohistochemistry was realized as in^10^. Nuclei were stained with 2 ug/ml Hoechst 33342 (Thermo-scientific #62249) and slides were mounted in Gelvatol pH = 8.5 (Sigma-Aldrich). For TUNEL assays frozen retinal sections (10 um) were incubated 1 h in blocking solution, and TUNEL assays was done following standard procedures from the manufacturer (*In Situ* Cell Death Detection Kit, Fluorescein, Roche #11684795910).

### Microscopy and imaging/quantification

Immunofluorescence microscopy (IFM) was conducted using an Axioplan microscope (Carl Zeiss Meditec, Thornwood, NY) equipped with a Spot RT-KE slider 7.4.1 camera (Diagnostic Instruments Inc., Sterling Heights, MI) and controlled by Spot 5.1 software and a Leica DM6000 widefield Fluorescence microscope equipped with a Hamamatsu Orca 03 G CCD camera (Hamamatsu Photonics K.K., Japan) and controlled by LAS X software. For confocal fluorescence microscopy, a Leica TCS SP5 II scanning laser confocal microscope (Leica Microsystems, Wetzlar, Germany) controlled by Leica Application Suite Advanced Fluorescence (LAS AF) software. Confocal images were captured using a 40x oil immersion objective (HCX PL APO CS, 1.25–0.75 NA).

For each IFM experiment presented, a minimum of 3 mice per genotype were captured, processed, analyzed using the same settings and quantified at identical threshold settings to reduce background fluorescence. An average of 4 regions of interest (500 μm length, central retina) per mouse were selected to quantify pHH3 and Pax6-positive nuclei. To quantify calretinin, calbindin, caspase-3, HuD, syntaxin, glutamine sythetase (GS) nuclei, an average of 2 regions of interest (500 μm length, central retina,) per animal were analyzed using ImageJ or Metamorph (Molecular Devices). To quantify GAD65, and AAK1 immunofluorescence, an average of 5 regions of interest were selected within the IPL (100 × 100 μm), the OPL (100 × 20 μm), and the IS (100 × 20 μm) in central retina and analyzed using Metamorph (Molecular Devices).

### RNA extraction and gene expression analyses

Total retinal RNA extraction was made from pools of 3 mice retinas following standard TRIzol procedures (Invitrogen-Life Technologies, Carlsbad, CA, #15596029) using pestle (USA Scientific Catalog no. 1415–5390) and DNase/RNase free zirconium beads (1.5 mm diameter, Benchmark, #D1032-30) for homogenization step and RNeasy mini kit (Qiagen, #74106). Total retinal RNA was eluted in DNase/RNase free water supplemented with RNaseOUT™ Recombinant Ribonuclease Inhibitor (40U/30uL) (Invitrogen, #10777-019), treated with RNase-free DNase and RNA concentrations and quality were assessed using Agilent Technologies 2100 Bioanalyzer, according to manufacturer protocols. Only RNA with RIN >8, A260/280 >1.9 and concentration >100 ng/uL was used. Strand-specific mRNA-seq libraries were prepared using TruSeq® mRNA Library Preparation Kits (Illumina, San Diego, CA), as described by manufacturer’s protocols^132–134^. The libraries were quantified (Kapa Biosystems assay) and sequenced on a NextSeq500/550 v2 chip (Illumina, San Diego, CA) to generate high-quality 75-bp reads (Q30 score >93%) with a depth of 30–60 million reads/sample.

### Bioinformatics analysis

RNA-Sequencing data were aligned to the mouse reference genome (RSubread). Data were normalized (Limma VOOM function) and differentially expressed genes (DEGs) were identified using linear modeling and Bayesian statistics (Limma)^135^. An in-house data analysis program provided by the PennVet Bioinformatics Core was used to average the data and identified the potentially transcriptionally induced and repressed genes in P28 mouse *Ndr2* KO retinas relative to WT controls. 341 potentially regulated genes (FC ≥ I2I) were classified into functional groups based on known gene ontology (GO) functions and pathways using the following online software and databases: Database for Annotation, Visualization and Integrated Discovery (DAVID), and Gene Ontology Consortium (GO).

To validate selected DEGs, RT-qPCR experiments were done in compliance with standard MIQE (Minimum Information for Publication of Quantitative Real-Time PCR Experiments) guidelines^136^. For RT-qPCR, RNA samples were reverse-transcribed using the High-Capacity cDNA Reverse Transcription Kit following standard procedures from the manufacturer (Applied Biosystems, Foster City, CA, #4368814). The RT-qPCR reactions contained 40 ng cDNA, 1x SYBR Green PCR Master Mix (Applied Biosystems, #4309155), and 250 nM of each unlabeled forward and reverse primer. Reactions were performed in 384-well reaction plate using the QuantStudio™ 6 Flex Real-Time PCR System (Applied Biosystems). GAPDH was found to be the most stable housekeeping gene in all tested samples, and used for normalization and calculation of the ratio of *Ndr* KO vs. WT using the ΔΔCT method^131,136^. Statistical significance of DEGs (p < 0.05; fold change (FC ≥ I2I) was assessed by one-sample T-test. Primers for RT-qPCR are listed in Supp. Table S3.

## Electronic supplementary material

Supplementary Tables

Supplementary Figures

## Data Availability

The datasets generated during and/or analyzed during the current study are available from the corresponding author on reasonable request.
