# Supplementary material for: Ndr kinases regulate retinal interneuron proliferation and homeostasis
Source: Sci Rep. 2018 Aug 22;8:12544. doi: 10.1038/s41598-018-30492-9 (PMC6105603; doi:10.1038/s41598-018-30492-9)
Supplement: Supplementary file 2 — Supplementary Figures [file 41598_2018_30492_MOESM2_ESM.pdf]

Supplemental Figures S1-S10 for:

Ndr kinases regulate retinal interneuron proliferation and homeostasis

Hélène Léger[1], Evelyn Santana[2], N. Adrian Leu[3], Eliot T. Smith[1], William A. Beltran[2], Gustavo D. Aguirre[2] and Francis C. Luca[1]

1. Department of Biomedical Sciences, University of Pennsylvania School of Veterinary Medicine, Philadelphia, PA, United States. 2. Division of Experimental Retinal Therapies, Department of Clinical Sciences and Advanced Medicine, University of Pennsylvania School of Veterinary Medicine, Philadelphia, PA, United States. 3. Center for Animal Transgenesis and Germ Cell Research, University of Pennsylvania School of Veterinary Medicine, Philadelphia, PA, United States.

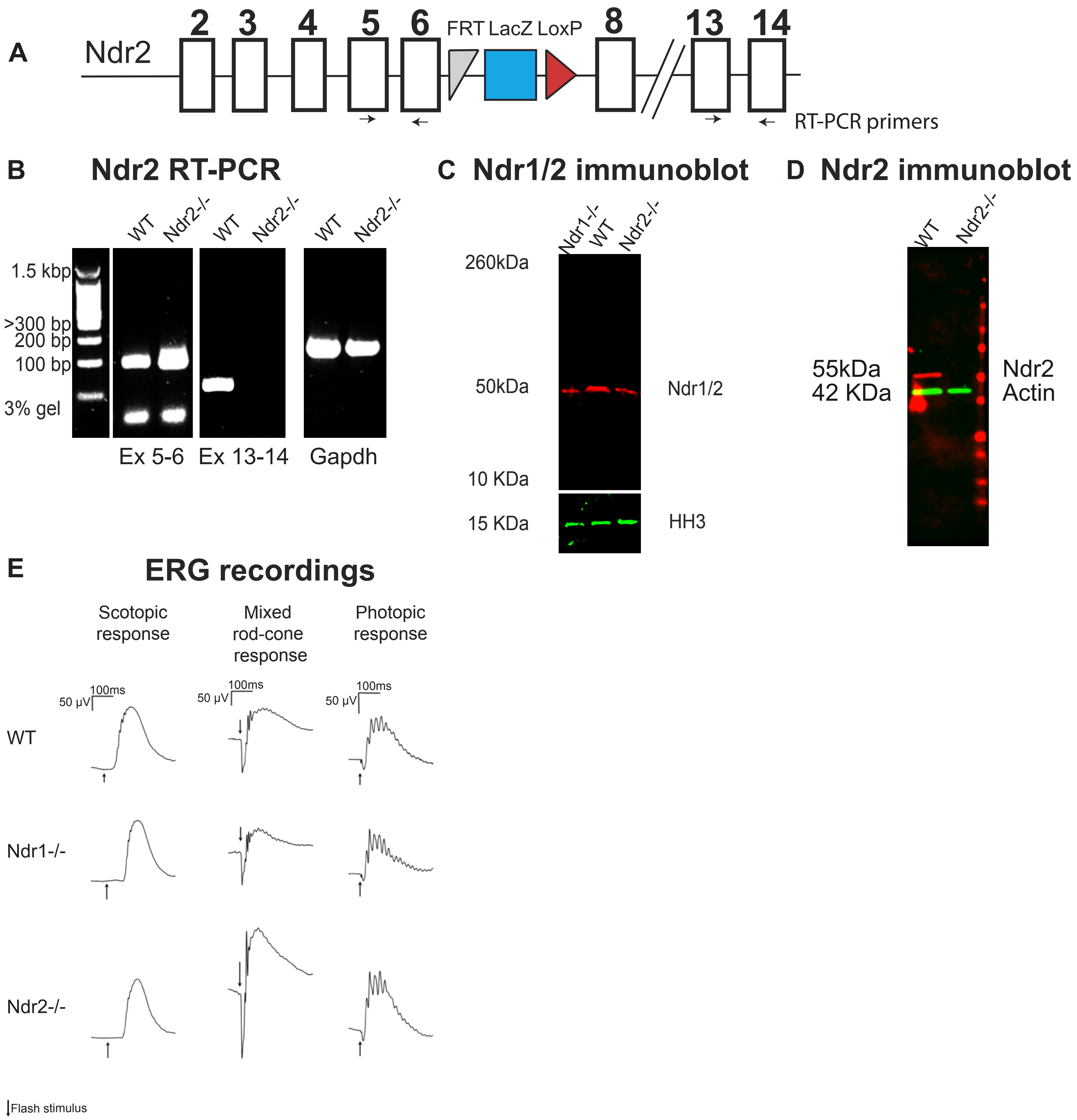

Fig. S1. Ndr2 KO validation and ERG recordings.

Léger et al.

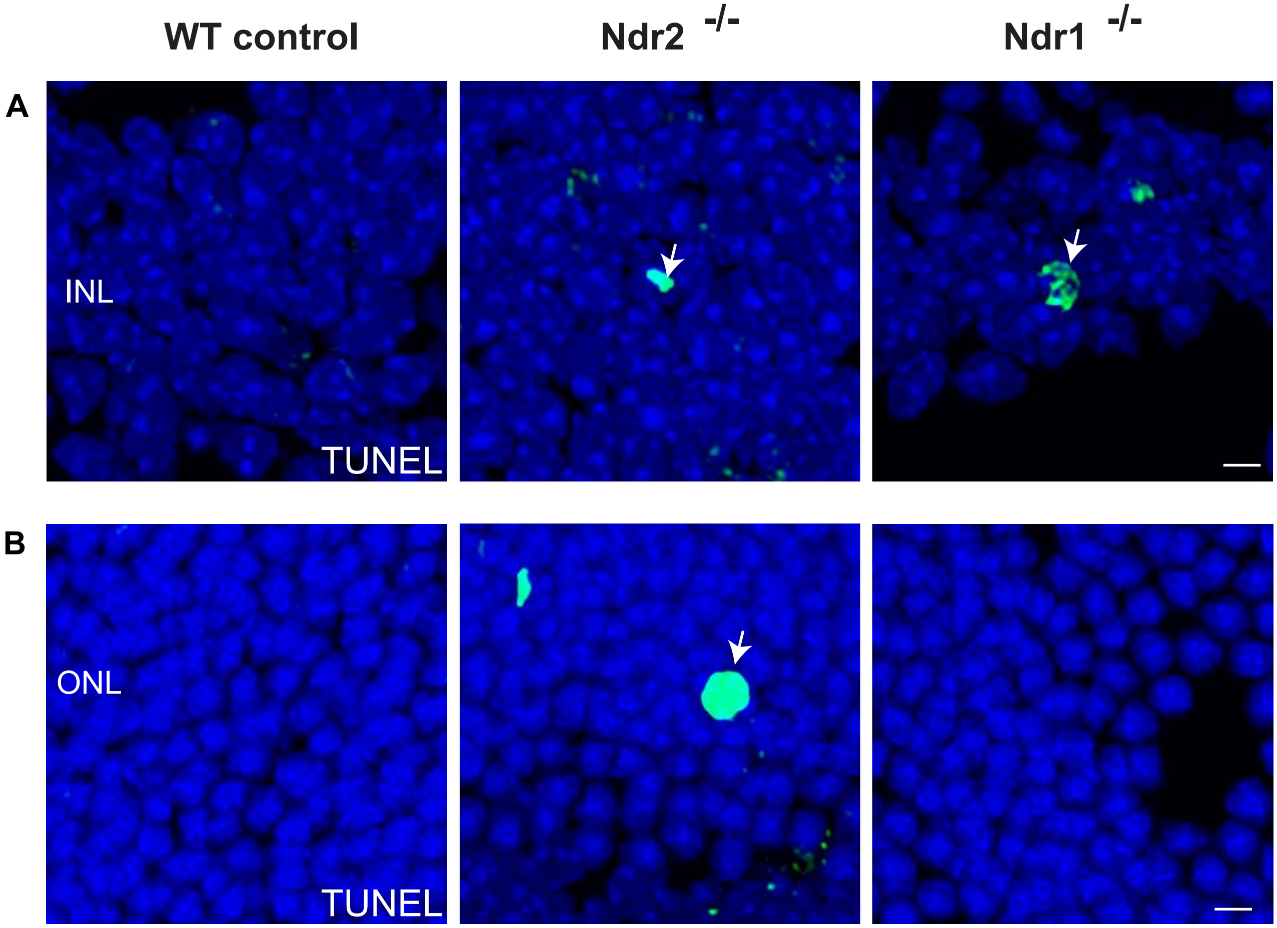

Fig. S2. Ndr deletion promotes cell death in the INL and ONL. Léger et al.

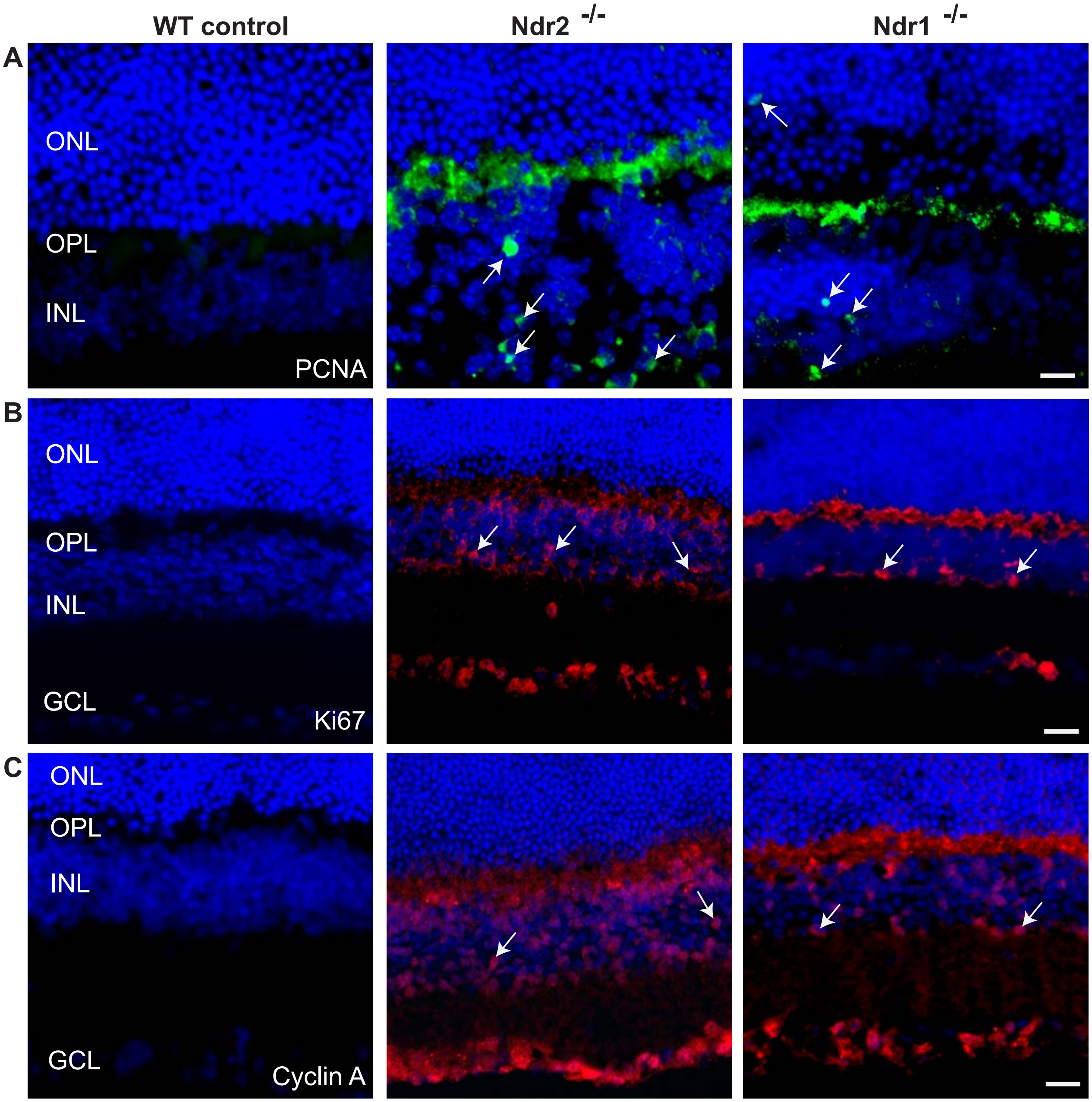

Fig. S3. Ndr deletion promotes cell proliferation in differentiated mouse retinas.
Léger et al.

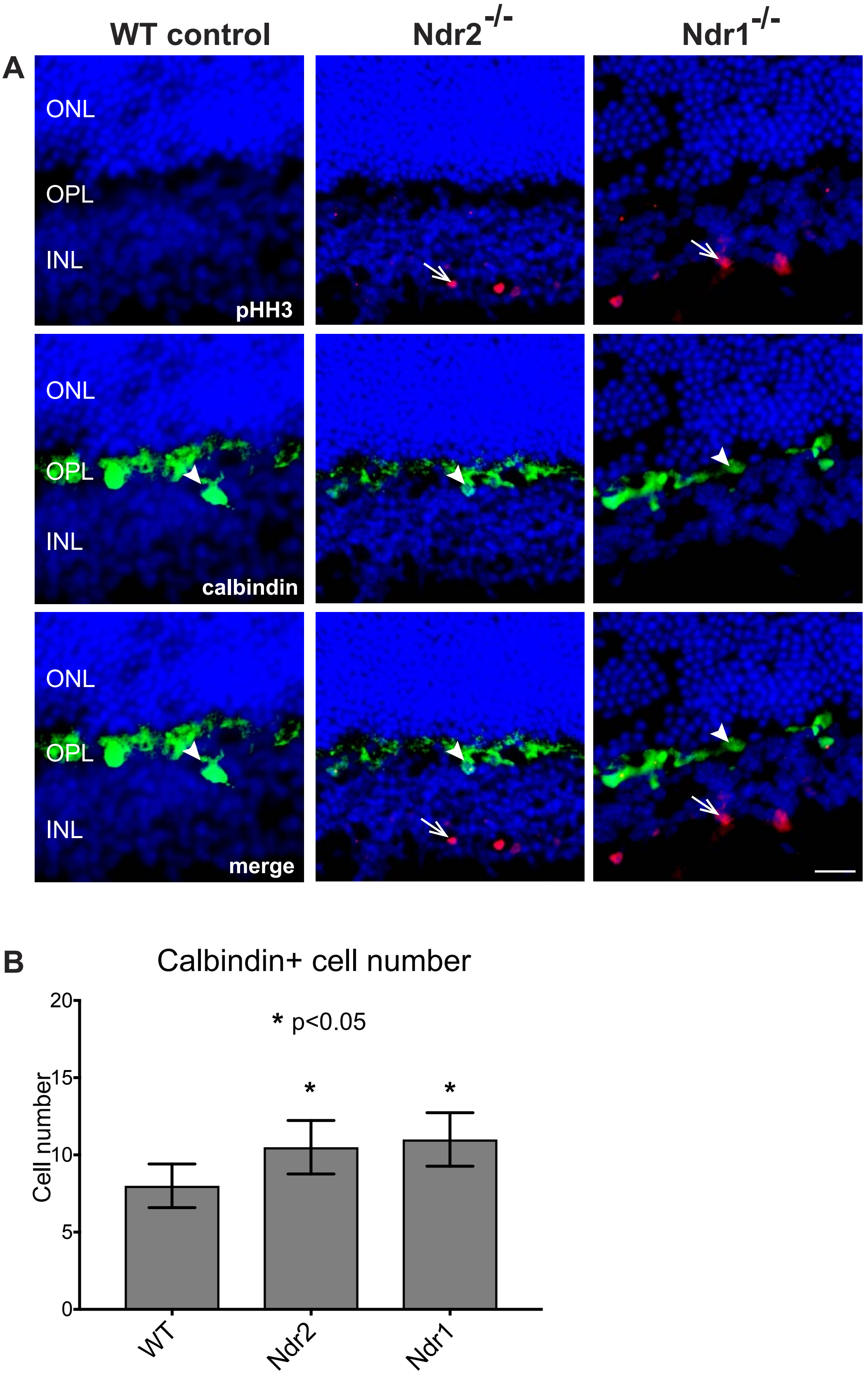

Fig. S4. Ndr deletion does not promote horizontal cell proliferation.
Léger et al.

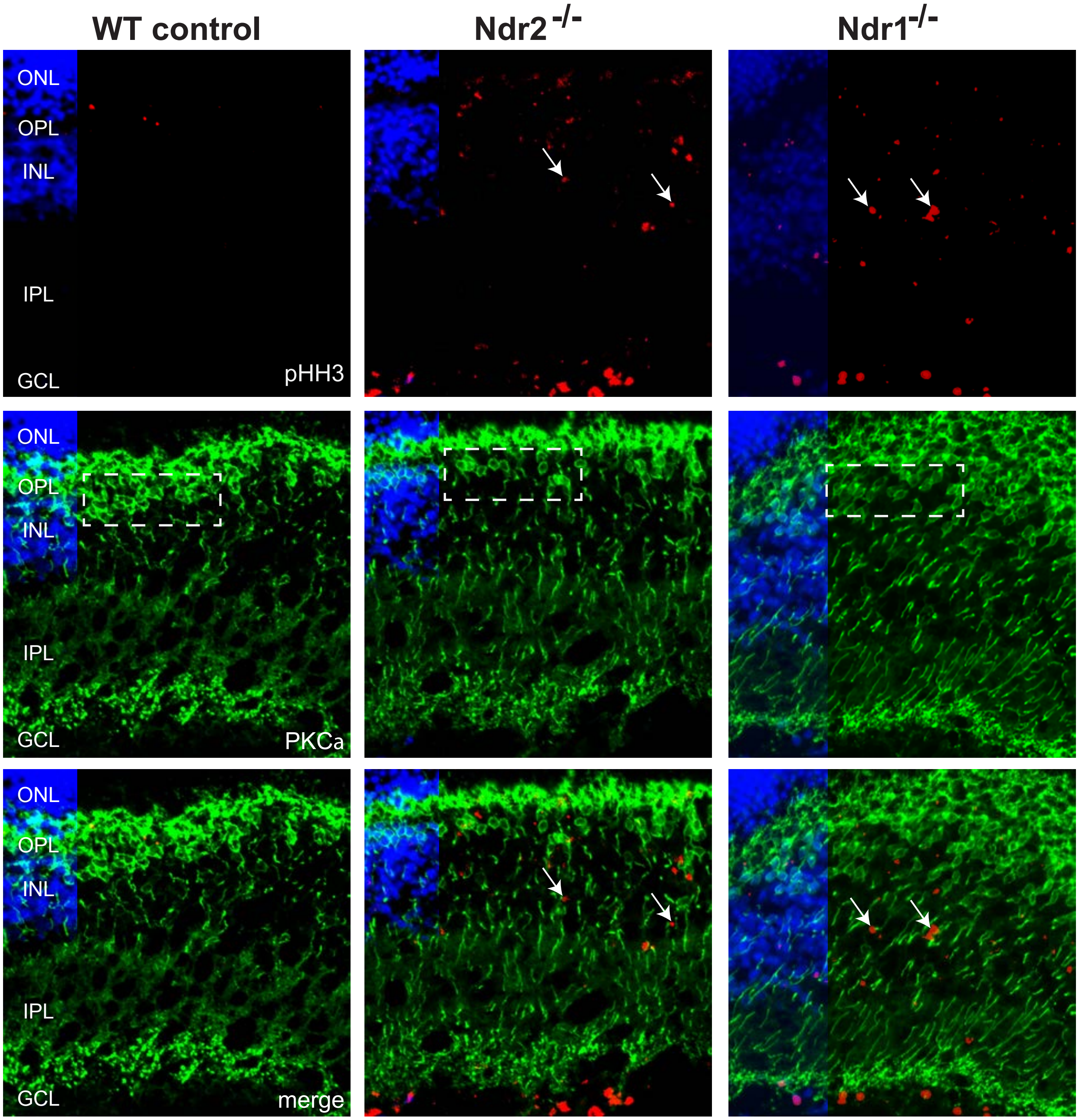

Fig. S5. Ndr deletion does not promote rod bipolar cell proliferation.
Léger et al.

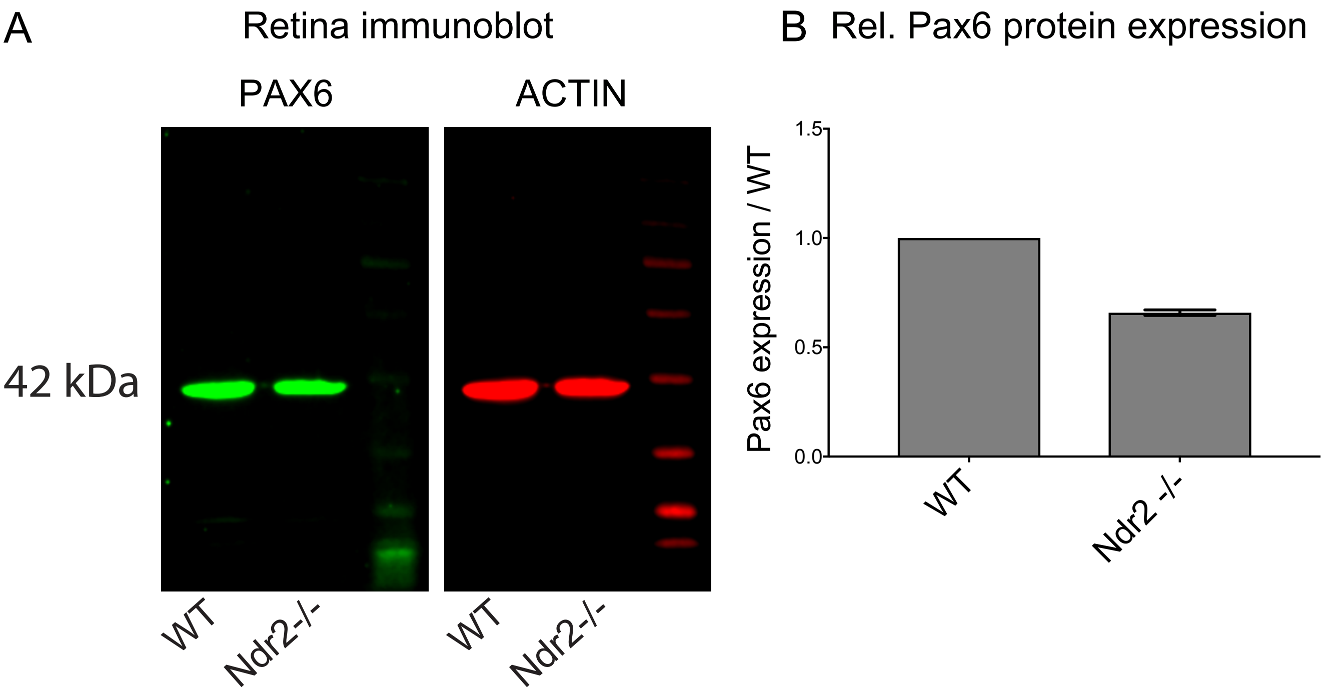

Fig. S6. Pax6 protein expression is diminished in Ndr2 KO retinas.
Léger et al.

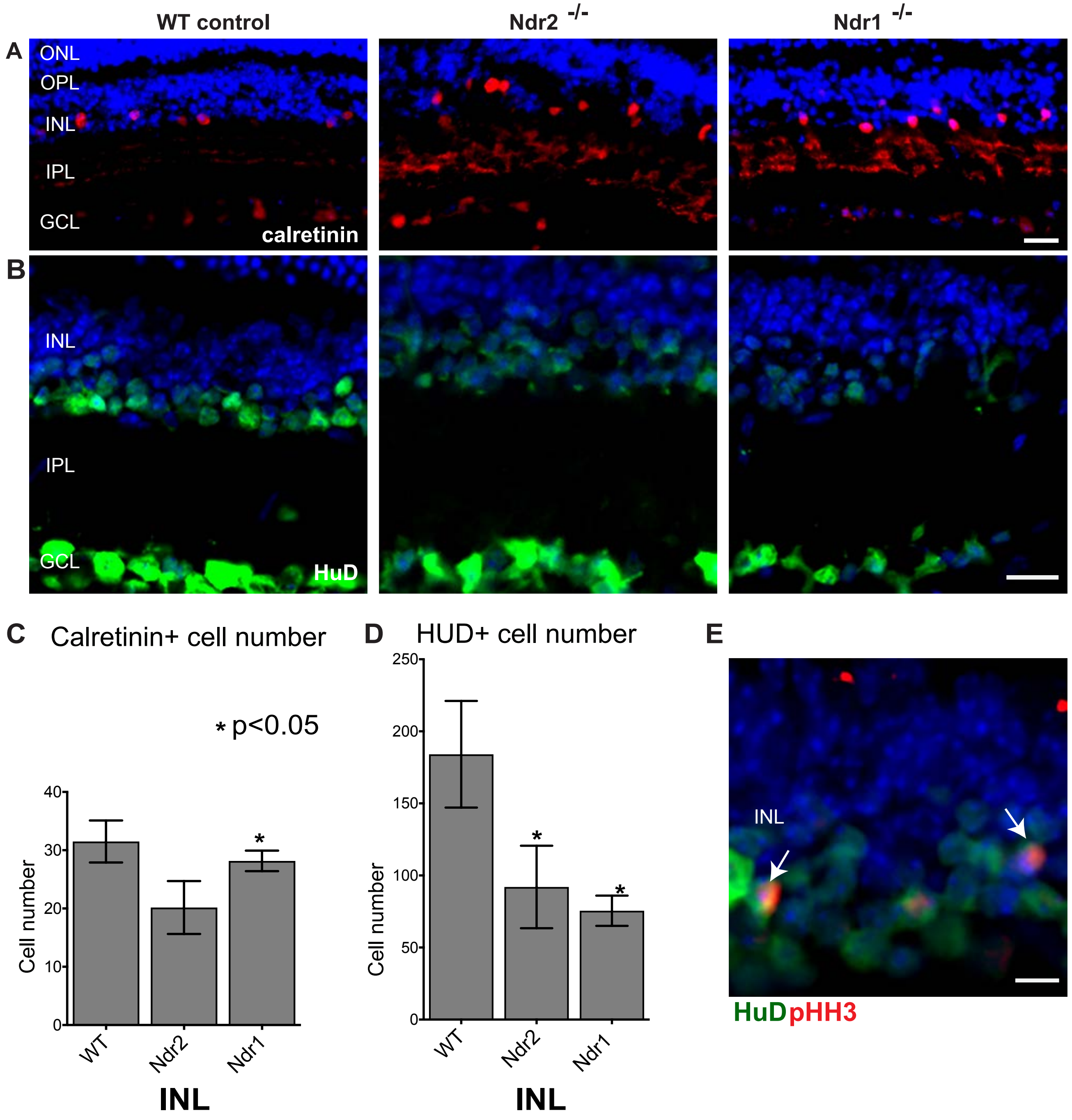

Fig. S7. Calretinin and HuD localization in Ndr KO retinas. Léger et al.

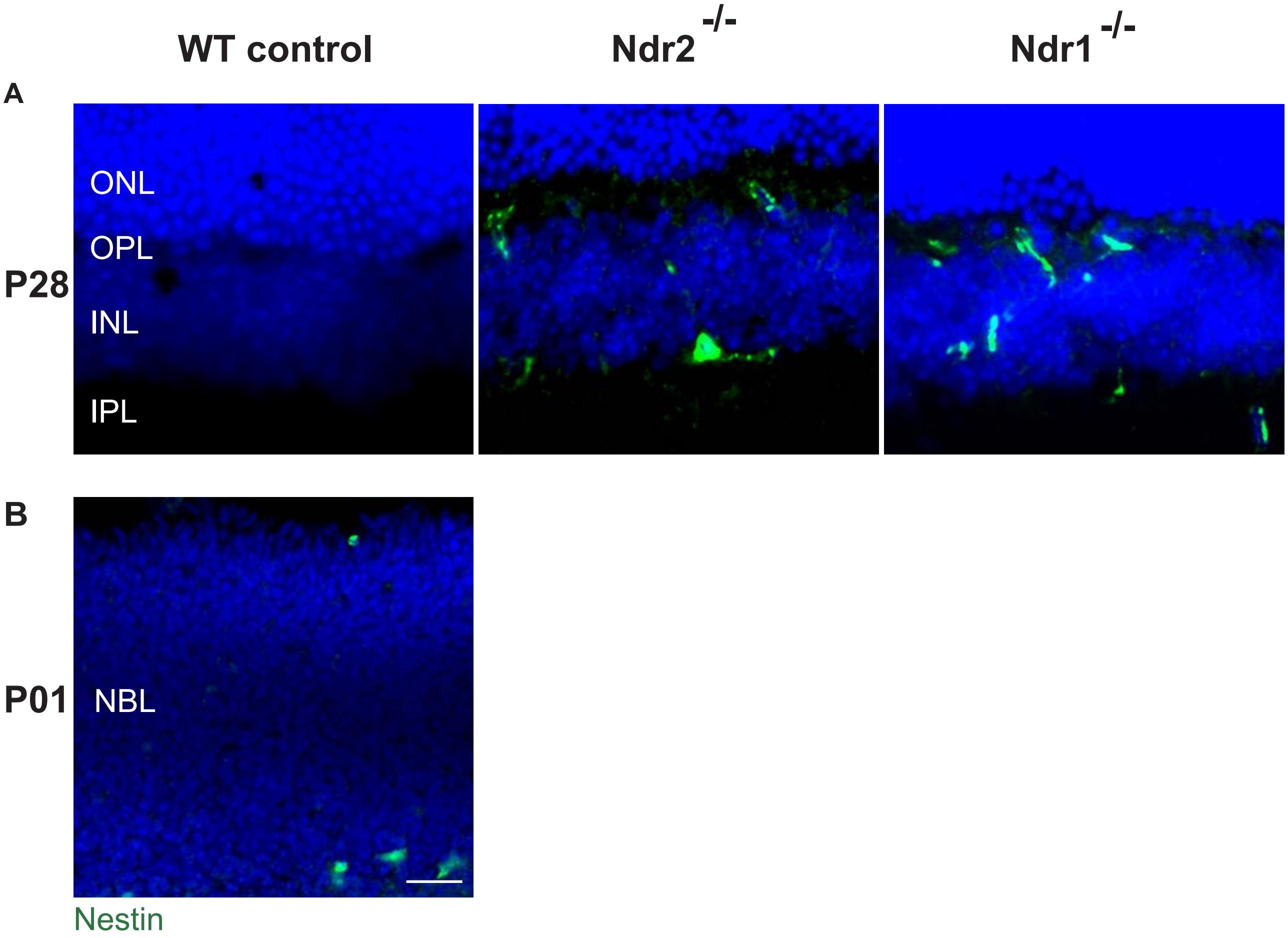

Fig. S8. Nestin localization in Ndr KO retinas.

Léger et al.

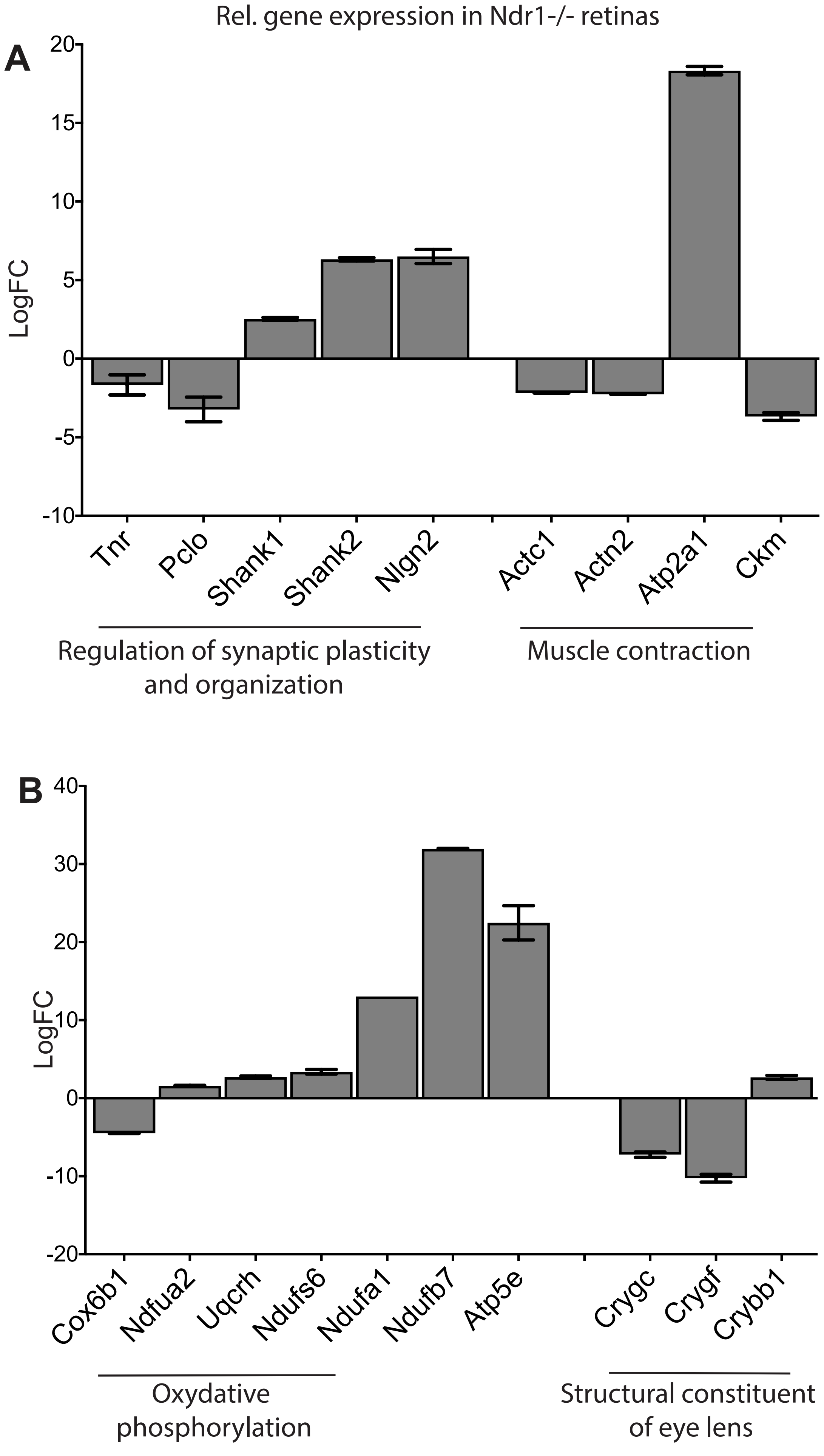

Fig. S9. mRNA expression in Ndr1 KO retinas. Léger et al.

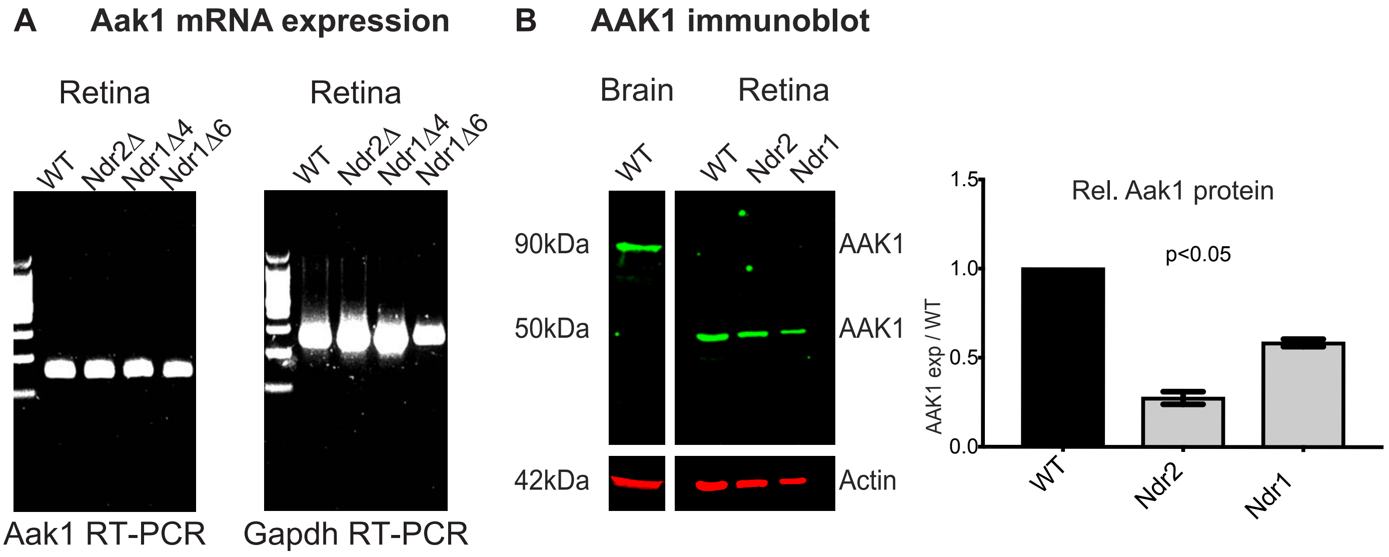

Fig. S10. Aak1 mRNA and protein expression in Ndr KO.

Léger et al.

Supplemental figure legends

Fig. S1. *Ndr2* KO validation and ERG response. (A) Diagram of the *Ndr2/Stk38l* conditional ready allele after exon 7 excision by cre recombinase. Arrows represent RT-PCR primers for amplifying Ndr2 transcripts containing exons 5-6 and 13-14. . (B) RT-PCR data show that Ndr2 transcripts are still present in exons 1 to 6 but absent in exons 13 to 14. cDNA was isolated from eye tissue from P28 wild type (WT) and *Ndr2* KO mice. GAPDH RT-PCR data serve as positive controls. (C) Immunoblot of brain protein extracts from P28 WT and *Ndr* KO mice probed with antibodies to Ndr1/2 (which was generated to the N-terminal 100 amino acids of Ndr1) and anti-Histone (HH3; 15 kDa). (D) Representative immunoblot of eye protein extracts from P28 WT and *Ndr2* KO mice probed with anti-Ndr2 (red) and actin (green) antibodies. Cropped images of this immunoblot are presented in Figure 1D.

(E) Representative ERG recordings from 1 month-old Ndr1 and Ndr2 KO mice performed at the Noninvasive Assessment of Visual Function facility (Penn Vision Research Center). The scotopic responses of dark-adapted animal measure rod and rod-driven bipolar cell response after a light flash (0.01 scot cd s m-2). The mixed rod-cone responses of dark-adapted animal measure the electric waves generated mostly by rods and rod-driven higher order retinal neurons after a bright flash (500 scot cd s m-2). The photopic responses of light-adapted (30 scot cd m-2 steady background light) measure the electric waves generated by cones and cone-driven higher order retinal neurons after a bright flash (500 scot cd s m-2). All ERG components are preserved in the Ndr KO mice. In addition, Ndr KO mice responded similarly to WT mice in visual placement assays (see methods).

Fig. S2. *Ndr* deletion promotes cell death in the INL and ONL. (A) TUNEL-positive cells (green) in INL (A) and ONL (B) from P28 *Ndr2* KO and *Ndr1* KO mice (see arrows). No

TUNEL-positive cells were detected in retinas WT mouse retinas. TUNEL-positive cells were rarely observed in the ONL in *Ndr1* KO mice (not shown). Images were acquired by confocal microscopy and visualized as a single optical section. Nuclei labeled with Hoechst 33342 (blue). Scale bars, 5 μM. INL, inner nuclear layer; OPL, outer plexiform layer; ONL, outer nuclear layer.

Fig. S3. *Ndr* deletion promotes cell proliferation in differentiated mouse retinas. (A) PCNA (green), (B) Ki67 (red)and (C) cyclin A (red) immunofluorescence of P28 WT and *Ndr* KO mouse retinas. PCNA-positive cells (green) are evident in the ONL and INL of *Ndr1* KO and INL of *Ndr2* KO retinas (arrows) and Ki67-positive and cyclin A-positive cells are present in the INL and GCL of *Ndr1 and Ndr2* KO retinas (arrows). Nuclei are labeled with Hoechst 33342 (blue). Scale bar, 20 μm.

Fig. S4. *Ndr* deletion does not promote horizontal cell proliferation. (A) pHH3 (red) and calbindin (green) immunofluorescence of retinal sections from P28 WT, *Ndr2* KO and *Ndr1* KO mice. DNA (blue). Scale bar, 20 μm. (B) The number of calbindin-positive cells from 500 μm length regions of INL are plotted ($n \geq 3$ mice per genotype). SD and significance were determined by unequal variance t-test ($p<0.05$).

Fig. S5. *Ndr* deletion does not promote rod bipolar cell proliferation. pHH3 (red) and protein kinase Cα (PKCα; green) immunofluorescence of retinas from P28 WT, *Ndr2* KO and *Ndr1* KO mice. The boxed regions indicate the location of rod bipolar cell bodies. Arrows denote representative pHH3-positive cells. Note that PKCα-positive rod bipolar cell bodies do not contain pHH3. Nuclei labeled with Hoechst 33342 (blue). Scale bar, 20 μm.

Fig. S6. Pax6 protein expression is diminished in *Ndr2* KO retinas. (A) Representative immunoblots of eye protein extracts from P28 WT and *Ndr2* KO mice probed with anti-Pax6 (green) and actin (red) antibodies using IRDye 680RD (red) IRDye 800CW (green) secondary antibodies. Fluorescence signals were simultaneously imaged using a LiCor Odyssey scanner. (B) The relative Pax6 levels were quantified from 2 independent experiments and plotted. SD and significance were determined by unequal variance t-test ($p<0.05$).

Fig. S7. Calretinin and HuD localization in *Ndr* KO retinas. (A) Calretinin (red) and (B) HuD (red) immunofluorescence of P28 WT and *Ndr* KO mouse retinas. Nuclei labeled with Hoechst 33342 (blue). Scale bar, 20 µM. The number of calretinin-positive (C) and HuD-positive (D) nuclei within 500 µm length regions of INL was plotted (n=3 mice). SD and significance were determined by one-way ANOVA test (*$p<0.05$). (E) Inset from Figure 6 showing representative HuD-positive (green) and pHH3-positive (red) cells from INL of Ndr2 KO retinas. Scale bar, 20 µM.

Fig. S8. Nestin localization in *Ndr* KO retinas. (A) Nestin immunofluorescence (green) in retinas from P28 WT and *Ndr* KO mice. (B) Nestin immunofluorescence in neuroblasts of P1 WT mice. . Nuclei labeled with Hoechst 33342 (blue). Scale bar, 20 µm.

Fig. S9. mRNA expression in *Ndr1* KO retinas. Comparative RT-qPCR of select genes in retinas from P28 *Ndr1* KO mice relative to WT. Genes chosen for analysis are from representative downregulated (A) and upregulated (B) genes identified in the *Ndr2* KO RNA-seq screen in Fig. 9 and 10. Histograms represent the log2 fold expression calculated as 2–ΔΔCt between Ndr1 KO and WT samples (log2FC≥ I1I). Means ± SD were calculated from a

minimum of 2 sets of RT-qPCR experiments with each sample run in duplicate ($p < 0.05$, calculated by one-sample test).

Fig. S10. Aak1 mRNA and protein expression in Ndr KO retinas. (A) RT-PCR reveal similar levels of Aak1 transcript in WT and Ndr KO retinas. cDNA was isolated from retinas from P28 wild type (WT), *Ndr2* KO and *Ndr1*$^{\Delta 4}$ KO mice. GAPDH RT-PCR data serve as positive controls. (B) Representative immunnoblots of brain and retina protein extracts from P28 WT, *Ndr2*and *Ndr1*$^{\Delta 6}$ KO mice were probed with antibodies to Aak1 (green) and actin (red). Relative Aak1 protein levels in Ndr KO retina extracts were quantified and plotted as ratios to WT. Similar data were obtained with two different Aak1 antibodies.

Supplemental Tables:

Sup. Table S1. Genotyping primers.

(A) Oligonucleotide primers for genotyping *Ndr2* KO mice; (B) Oligonucleotide for *Ndr1* synthetic single-guide RNA (sgRNA) construction: All sgRNA were designed with a T7 promoter for in vitro transcription (bold), the 20 base spacer target specific (black), and an overlapping region that anneals to the constant oligonucleotide (underlined) and ordered as a 60 base oligonucleotide **TAATACGACTCACTATA**-N20-<u>GTTTTAGAGCTAGAAATAGCAAG</u>. The 80 bases constant oligonucleotide was simultaneously ordered; (C) Oligonucleotides for High Resolution Melt (HRM) qPCR and DNA sequencing; (D) Oligonucleotide primers for genotyping *Ndr1* KO mice. KASP oligos for *Ndr1*$^{\Delta 4}$ were designed and provided by LGC laboratory (Ndr1_i3e4ie_M2, project 1773.24).

Sup. Table S2. Antibodies. List of antibodies used for immunohistochemistry (IHC) and western blot (WB). Antibodies are reported with the symbol of the corresponding protein

(antigen), source including commercial company name (catalogue number) and concentrations used for either IHC or WB.

Sup.Table S3. List of RT-PCR and RT-qPCR primers. RT-qPCR primers are reported with the symbol of the corresponding gene.

Sup Table S4. Comparative RNA-seq data from *Ndr2* KO retinas. DEGs with an absolute value of >2 fold change (log2FC≥ I1I).

Sup. Table S5. Gene ontologies from *Ndr2* KO RNA-seq data. (A) Molecular functions; (B) Biological process (FC≥ I2I).

Sup. Table S6. Top up and down regulated genes from *Ndr2* KO RNA-seq data analysis. DEGs with an absolute value of >3 fold change (log2FC≥ I1.5I).
